# The consensus-based CINEX guideline for reporting clinical information extraction studies

**DOI:** 10.1093/jamia/ocag136

**Published:** 2026-08-08

**Authors:** Daniel Reichenpfader, Jamil Zaghir, Elsa Cécilia-Joseph, Boris Delange, Anastassia Shaitarova, Emmanuelle Sylvestre, Fabio Dennstädt, Boya Zhang, Douglas Teodoro, Stéphane Meystre, Henning Müller, Kerstin Denecke

**Affiliations:** School of Life Sciences, Faculty of Medicine, University of Geneva, Geneva, 1206, Switzerland; Institute for Patient-Centered Digital Health, Bern University of Applied Sciences, Biel, 2502, Switzerland; Division of Medical Information Sciences, Geneva University Hospitals, Geneva, 1202, Switzerland; Department of Radiology and Medical Informatics, University of Geneva, Geneva, 1202, Switzerland; Université de Rennes, CHU Rennes, INSERM, LTSI—UMR 1099, Rennes, F-35000, France; CHU Martinique, Centre de Données Cliniques, Martinique, F-97200, France; Université de Rennes, CHU Rennes, INSERM, LTSI—UMR 1099, Rennes, F-35000, France; Department of Computational Linguistics, University of Zurich, Zurich, 8050, Switzerland; Université de Rennes, CHU Rennes, INSERM, LTSI—UMR 1099, Rennes, F-35000, France; CHU Martinique, Centre de Données Cliniques, Martinique, F-97200, France; Department of Radiation Oncology, Inselspital, Bern University Hospital and University of Bern, Bern, 3010, Switzerland; Faculty of Medicine, University of Geneva, Geneva, 1211, Switzerland; Department of Radiology and Medical Informatics, University of Geneva, Geneva, 1202, Switzerland; Institute of Digital Technologies for Personalised Healthcare (MeDiTech), University of Applied Sciences and Arts of Southern Switzerland (SUPSI), Lugano, 6962, Switzerland; Department of Radiology and Medical Informatics, University of Geneva, Geneva, 1202, Switzerland; Informatics Institute, HES-SO Valais-Wallis, Sierre, 3960, Switzerland; Institute for Patient-Centered Digital Health, Bern University of Applied Sciences, Biel, 2502, Switzerland

**Keywords:** information extraction, reporting guideline, reporting quality, Delphi study, consensus statement

## Abstract

**Objective:**

Information extraction (IE) from clinical texts has advanced rapidly with recent advances in natural language processing, particularly the advent of large language models (LLMs). However, inconsistent and incomplete reporting of methodologies limits reproducibility, comparability, and clinical translation. We aimed to develop a consensus-based reporting guideline tailored to clinical IE studies.

**Material and Methods:**

We developed the Clinical Information Extraction Reporting Guideline (CINEX) through a multi-phase process. The initiative was prospectively registered on the EQUATOR Network as a reporting guideline under development, and a detailed Delphi study protocol was published in advance. A scoping review informed an initial set of items, which was refined through a 3-round electronic Delphi study with 20 international experts, followed by a final consensus meeting. Items were iteratively refined based on predefined inclusion criteria and expert feedback.

**Results:**

The final CINEX guideline comprises 29 checklist items grouped into 5 domains: information model, architecture, data, annotation, and outcomes. The 3 eDelphi rounds included 20, 15, and 12 experts, respectively. Two items were added after round one. Consensus for inclusion was reached for a 21 and additional 7 items after rounds 2 and 3, respectively.

**Discussion:**

CINEX provides a structured framework to improve transparency, reproducibility, and interpretability in clinical IE research. By standardizing reporting of key methodological components, such as data provenance, annotation processes, and evaluation strategies, it facilitates meaningful comparison across studies and supports safer clinical implementation.

**Conclusion:**

CINEX complements existing AI reporting standards by addressing domain-specific challenges in clinical IE.

## Introduction

Physicians and nurses spend approximately 37% and 22% of their working time interacting with the electronic health record (EHR).^1^ For clinicians, the time needed to look up information exceeds the time creating new documentation.^1^ In primary care, the time needed for documentation might even surpass the visit duration itself.^2^ Because substantial clinical effort is already invested in creating and reviewing EHR text, the ability to extract structured information from these documents is increasingly important for downstream use. This is the domain of clinical information extraction (IE), a subfield of natural language processing (NLP). Clinical IE aims to transform unstructured documentation into structured data for further reuse.

In this work, we adopt the definition proposed by Okurowski^3^ for IE as the task of extracting instances of specific, pre-defined generic information types from unstructured text.^4^ This definition therefore comprises the subtasks of document-level classification, named entity recognition (NER), relation extraction (RE), template filling, and (extractive) question answering, but excludes extractive and abstractive summarization. According to Jurafsky and Martin,^5^ these subtasks are implemented using heuristics, machine learning, or deep learning methods. With the advent of the transformer-based deep learning architecture, the potential of clinical IE first increased substantially in 2017,^6^ and subsequently experienced increased public and professional attention with the publication of ChatGPT, the first commercial LLM service based on the GPT-3.5 model, provided by OpenAI in November 2022.^7^

LLMs are “deep learning models with a huge number of parameters trained in an unsupervised way on large volumes of text”.^8^ These models have demonstrated improved performance for various healthcare applications, including medical question answering,^9^^,^^10^ and clinical text summarization.^11^ Specifically, there is growing evidence that LLMs are capable of extracting structured information from clinical text.^12^^,^^13^ However, technological progress is rapid and continuous, with new model architectures, training methods, and evaluation benchmarks emerging weekly.

To keep pace with these developments and to synthesize evidence on key aspects such as performance, safety, and clinical utility, comparability across studies is essential. Transparent reporting of system design, data sources, annotation workflows, and evaluation metrics is critical for fair comparison, reproducibility, and eventual translation into clinical practice. For clinicians, such transparency is equally important: without clarity on how an IE system was built and validated, its clinical suitability, risks, and expected benefits remain uncertain. This problem is aggravated by currently insufficient evaluation methodologies of clinical LLM applications that fail to capture real-world efficacy.^14^

Despite this need, reporting practices in clinical IE research are highly heterogeneous, making it difficult to interpret findings, compare approaches, or reproduce results. Existing artificial intelligence (AI)-focused reporting guidelines such as SPIRIT-AI,^15^ CONSORT-AI,^16^ TRIPOD-AI,^17^ and TRIPOD-LLM^18^ provide guidance for prediction modeling and interventional AI studies, but they do not fully address the unique methodological challenges of clinical IE, such as the transparent specification of the information model or annotation process as shown by their coverage of CINEX-defined IE reporting elements in Table 1. Journal-specific guidelines for reporting of AI-related healthcare applications are similarly phrased too broadly.^19^ A systematic review and meta-analysis on reporting guidelines in medical AI by Kolbinger et al^20^ highlights the importance of reporting guidelines in clinical AI research and underscores the need for common standards that address the identified variations and gaps in current guidelines”.

**Table 1.** Coverage of CINEX-defined clinical IE reporting elements by existing reporting guidelines.

Dimension/item	CINEX	**SPIRIT-AI** ^15^	**CONSORT-AI** ^16^	**TRIPOD-AI** ^17^	**TRIPOD-LLM** ^18^
**Information model**					
**1. Details of extracted information types**	⚫	○	○	▲	▲
**2. Underlying clinical information model**	⚫	○	○	○	○
**3. Data model**	⚫	○	○	○	○
**4. Semantic aspects of the extraction process**	⚫	○	○	○	▲
**5. Complementing aspects of the extraction process**	⚫	○	○	○	○
**Architecture**					
**6. Task**	⚫	▲	▲	▲	⚫
**7. Implementation**	⚫	▲	▲	▲	▲
**8. Pre-processing**	⚫	▲	▲	⚫	⚫
**9. Hyperparameters**	⚫	▲	▲	▲	▲
**10. Hardware details**	⚫	○	○	○	⚫
**Data**					
**11. Data flow**	⚫	▲	▲	⚫	⚫
**12. Splitting strategy**	⚫	▲	▲	⚫	⚫
**13. Origin**	⚫	⚫	⚫	⚫	⚫
**14. Type**	⚫	○	○	▲	▲
**15. Time frame**	⚫	▲	▲	⚫	⚫
**16. Representativeness and imbalance measures**	⚫	○	○	⚫	⚫
**17. Examination and medical specialty**	⚫	○	○	○	○
**18. Language**	⚫	○	○	○	⚫
**19. Ethical approval**	⚫	⚫	▲	⚫	⚫
**Annotation**					
**20. Annotation approach**	⚫	○	○	○	⚫
**21. Annotation process**	⚫	○	○	○	⚫
**22. Annotation guidelines**	⚫	○	○	○	⚫
**23. Annotation tools**	⚫	○	○	○	○
**24. Inter-annotator agreement**	⚫	○	○	○	▲
**Outcome**					
**25. Performance**	⚫	▲	⚫	⚫	▲
**26. Statistical validation**	⚫	⚫	⚫	⚫	▲
**27. External validation**	⚫	○	▲	▲	▲
**28. Data availability**	⚫	⚫	○	⚫	⚫
**29. Source code availability**	⚫	▲	○	⚫	⚫

⚫: Full coverage (explicitly addressed); ▲: Partial coverage; ○: Not addressed.

The Clinical Information Extraction Reporting Guideline (CINEX), presented in this paper, addresses these gaps. Developed through a consensus-based process, CINEX defines minimum reporting requirements tailored to clinical IE research, harmonizes key terminology, and promotes open science practices. Its goal is to support researchers, clinicians, and developers alike in publishing transparent, comparable, and clinically meaningful IE studies. Therefore, CINEX contributes to advancing the safe and effective integration of clinical IE technologies into healthcare systems.

## Methods

### Overview

The CINEX development process was prospectively registered with the EQUATOR Network as a reporting guideline under development in October 2024 (https://www.equator-network.org/library/reporting-guidelines-under-development/reporting-guidelines-under-development-for-other-study-designs/#CINEX). An executive committee (DR, HM, KD) coordinated all stages of development. The committee ensured adherence to the methodological guidance for reporting guideline development proposed by Moher et al^21^ and oversaw all activities related to data collection, analysis, and consensus building.

As shown in Figure 1, CINEX was developed in 3 main phases: (1) identification of the need for the guideline, (2) preliminary guideline development, and (3) finalization through consensus building and consolidation in the course of an electronic Delphi (eDelphi) study and a final consensus meeting.

**Figure 1.**
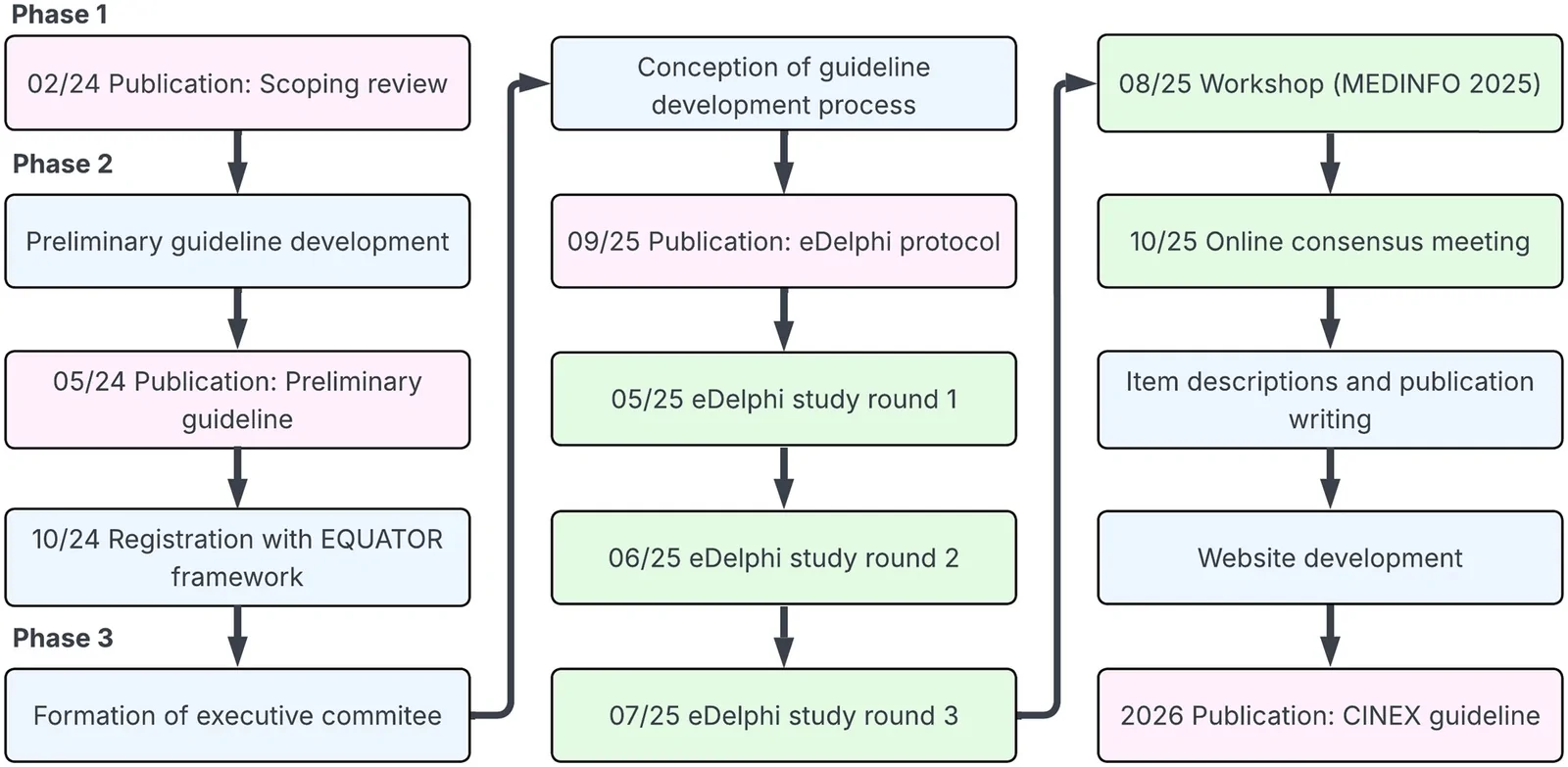
Overview of the CINEX development activities. Red: Publications. Green: eDelphi-related activities. Blue: Other activities.

#### Phase 1: Identification of need

The need for a dedicated reporting guideline targeting clinical IE was identified through a scoping review of clinical IE studies focusing on radiology reports.^22^ This review identified prevailing reporting practices, methodological gaps, and challenges in transparency. A structured data extraction table captured key study characteristics, IE methods, datasets, annotation procedures, evaluation metrics, and transparency indicators.

#### Phase 2: Preliminary guideline development

Drawing on the findings of the scoping review, we developed an initial set of 28 candidate reporting items grouped into 5 preliminary domains, including information model, system architecture, data, annotation, and outcomes. Items were phrased to be technology-agnostic and applicable to both rule-based and machine learning approaches.^4^

#### Phase 3: Consensus and finalization

The finalization phase aimed to refine and validate the preliminary CINEX items through expert consensus. This phase included a 3-round electronic Delphi (eDelphi) study followed by a consensus meeting and is described in detail in the next section.

### eDelphi study design and participants

The eDelphi study was conducted between May and July 2025, following methodological principles by Häder,^23^ Nasa et al,^24^ and Trevelyan and Robinson,^25^ as outlined in the study protocol.^26^ The consensus development process was reported in accordance with the ACCORD (ACcurate COnsensus Reporting Document) guideline;^27^ the completed ACCORD checklist is provided as Supplementary Material. Eligible participants included domain experts in clinical IE recruited via publication search and the executive committee’s professional networks. The eDelphi panel included coauthors of the manuscript as voting panelists, as well as additional external experts who were not part of the author group. A total of 20 experts were invited to and participated in round one, yielding 19 valid responses. In round 2, the 19 panelists with valid round-one responses were invited, of whom 15 provided valid responses (78.9%). In round 3, the 15 panelists with valid round-2 responses were invited, of whom 12 provided valid responses (80.0%). All surveys were administered using the locally hosted, open-source survey platform LimeSurvey.^28^ The exported survey instruments are available in the OSF repository: https://doi.org/10.17605/OSF.IO/ATSH9. The eDelphi study was reviewed by the institutional (Bern University of Applied Sciences) ethics committee and was determined to be exempt from formal ethics approval. All participants provided informed consent before participating in the survey, including consent for the anonymized use and publication of their free-text comments.

Participants rated each reporting item on two 10-point scales assessing *relevance* and *maturity* (1 = not at all relevant/mature; 10 = very relevant/mature). Consensus for inclusion was defined as a mean score ≥8 with a SD ≤2 for both dimensions. Items with mean ≤3 and SD ≤2 were excluded. Items outside these thresholds were revised and reevaluated in subsequent rounds. The objective of round one was to assess the relevance and maturity of the preliminary reporting items and to identify missing items. The objective of round 2 was to reevaluate revised items and newly proposed items after structured feedback from round one. The objective of round 3 was to obtain final ratings on items that had not yet reached consensus after round 2.

After each round, participants received aggregated feedback in the form of histograms, anonymized comments, and their own prior ratings to support reflection and consistency across rounds. New items could be proposed during the first round, and rephrasing was applied iteratively based on panel feedback. For the final consensus meeting, consensus was defined as ≥80% agreement among participants, with the meeting considered quorate if at least 50% of round-1 panelists were present.

## Results

### Results of the eDelphi study

Among the 20 experts who participated in round one, 15 were male and 5 were female. The participants represented 7 countries: Switzerland (*n* = 9), France (*n* = 4), the United States (*n* = 3), Austria (*n* = 1), Brazil (*n* = 1), China (*n* = 1), and Germany (*n* = 1). In terms of expertise, none declared themselves as novice or basic, while 4 participants were at the intermediate level, 10 at the advanced level, and 6 at the expert level, with years of experience ranging from 2 to 23 years (average: 8.1 years, median: 5.5 years).

After the first eDelphi round, 2 items were added to the preliminary set of 28 items. The majority of items (*n* = 23) were revised based on the panelists’ comments. After the second round, 21 items reached consensus for inclusion and 5 more items were revised. The third round resulted in an additional 7 items reaching consensus. During all 3 rounds, no item achieved consensus for exclusion. Two items did not reach consensus (ie “Data—Anatomical region” and “Architecture—Hardware details”). Across rounds, non-consensus was driven mainly by the maturity dimension rather than by relevance: maturity ratings showed higher variability than relevance ratings for several items. Panel comments suggested that maturity was more dependent on context, including local infrastructure, national regulations, and differences in clinical documentation practices.

Following the 3 eDelphi rounds, an in-person feedback meeting was held as a workshop at the MEDINFO 2025 conference in August 2025 to collect additional feedback for guideline improvement. Participants suggested including the clinical purpose of the extraction project into the guideline as well as explicit naming of the items in the annotation section (eg, “Annotation approach” instead of “Approach”). The final online consensus meeting was held in October 2025 and included 12 participants who discussed the last 2 items that had not achieved agreement during the eDelphi panel. The item “Hardware details” reached consensus after resolving ambiguities in its description. The item “Anatomical region” was removed and folded into the item “Type.” Furthermore, the feedback of the MEDINFO workshop was implemented by explicitly naming 4 items of the annotation dimension and agreeing to elaborate the clinical purpose in the elaboration of the item “Underlying clinical information model.” In summary, the preliminary set of 28 items was expanded by 2 newly proposed items after round one; 21 items reached consensus after round 2 and 7 additional items after round 3, while the remaining 2 items were resolved in the final consensus meeting by including “Hardware details” and folding “Anatomical region” into “Type,” resulting in 29 final CINEX items. See Figure 2 for an overview of the evolution of items across rounds. The eDelphi survey instruments and item-level quantitative data, including round-by-round relevance and maturity means, SDs, item evolution, and panel comments, are available in the OSF repository: https://doi.org/10.17605/OSF.IO/ATSH9.

**Figure 2.**
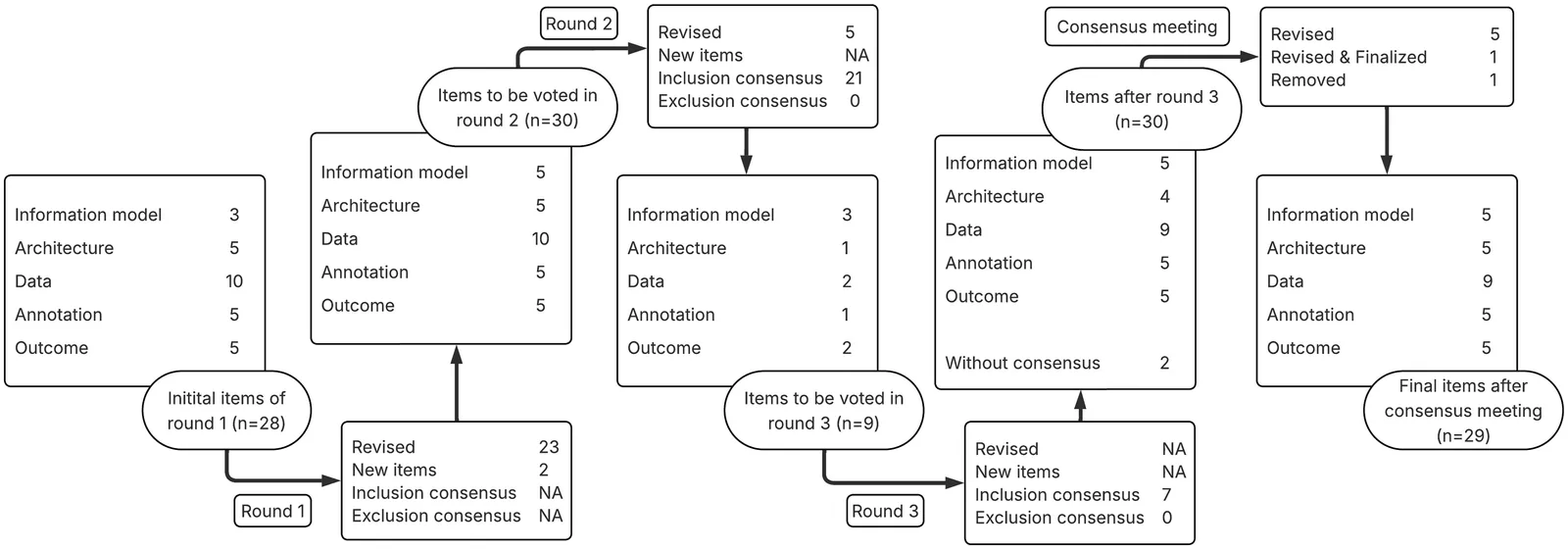
Item evolution during the eDelphi study.

### The CINEX guideline

The final CINEX guideline consists of 5 main domains (information model, architecture, data, annotation, and outcome) and 29 checklist items (Table 2). Each item represents a minimum reporting requirement. In the explanation and elaboration document, we provide a rationale, key elements to address, and concrete examples for each item, see Supplementary Material.

**Table 2.** The CINEX guideline, comprising 29 reporting items across 5 dimensions.

Dimension/item	Item description
**Information model**	
**1. Details of extracted information types**	Clearly list and define each type of clinical information the algorithm is designed to extract, such as entities (medications, diagnoses, procedures, or laboratory values), relations, or document classes. Provide examples for each type, especially when extracting complex, multi-part types and/or concepts.
**2. Underlying clinical information model**	Describe the underlying clinical information model or clinical framework used to guide the structuring or interpretation of extracted data, if applicable. Furthermore, justify the choice of model and its context. Describe any adaptations or assumptions made and limitations of the information model.
**3. Data model**	Describe the underlying data model used to guide the structuring or interpretation of extracted data, if applicable.
**4. Semantic aspects of the extraction process**	Explain how extracted entities are organized by mapping to terminologies, ontologies, or thesauri.
**5. Complementing aspects of the extraction process**	If applicable, describe how uncertainty, negation, temporality, ambiguity, inconsistency, and coreference resolution (eg, patient vs family member) is handled.
**Architecture**	
**6. Task**	Describe the primary information extraction task(s), as well as additional steps performed as part of the study, such as document classification, named entity recognition (NER), relation extraction, template filling, or summarization. For complex workflows (eg, agentic behavior, multi-step pipelines), describe each step in detail.
**7. Implementation**	Detail the implementation strategy used to develop the algorithm, including any specific frameworks, pipelines, or approaches. When using existing software, include version specifiers and parameters. If applicable, depict the complete machine learning system architecture including its components. For LLMs, provide the model card.
**8. Pre-processing**	Describe text normalization or cleaning steps performed prior to model input, including the rationale and details of the approach taken. When using existing software, include version specifiers and parameters.
**9. Hyperparameters**	List all hyperparameters used during model training and provide their values and selection process (manual, grid search, etc.). For in-context learning, provide the exact prompts used and describe the prompt development process.
**10. Hardware details**	Report hardware specifications (eg, GPU device name, vRAM, cluster description, GPU hours) of training and inference environments and costs, if applicable and available.
**Data**	
**11. Data flow**	Provide a detailed description of the data processing workflow, to report on the processing of selecting, excluding, filtering, and sampling data from the originally available pool of documents and on the number of resulting documents/sentences/words/tokens for each step. The provision of a visualization is complementary.
**12. Splitting strategy**	Show and explicitly describe the applied data splitting strategy (train, test, evaluation, and/or validation set) including the number of documents/sentences/words/tokens, splitting modality (timeline, groups, etc), and method (random, stratified).
**13. Origin**	Identify the department(s), institution(s), and country(ies) from which the clinical texts originated. If applicable, describe which (clinical) information system data was extracted from.
**14. Type**	Describe the clinical text source (author type, stage in patient treatment where it is created (eg, at discharge, during stay) and their purpose (eg, progress monitoring), and content (eg, daily insights from nurses).
**15. Time frame**	Describe the time frame and (binned) time-related distribution of the dataset.
**16. Representativeness and imbalance measures**	Describe the population the data used in the study might be representative of, including any limitations. If applicable, report the class distribution on all levels (source, sample, split) and any methods used to address class imbalance.
**17. Examination and medical specialty**	If applicable, describe the examination and medical specialty the source document corresponds to.
**18. Language**	Specify the language(s) of the clinical texts used for algorithm development and evaluation. Describe any manipulation of the original data (eg, translation).
**19. Ethical approval**	State whether the study received ethics committee approval and provide the approval reference number if applicable.
**Annotation**	
**20. Annotation approach**	Describe whether the textual annotation method is manual, rule-based, machine learning-based, or hybrid (ie, if automated annotation was combined with manual annotation), including its sequence. Describe the applied tagging format, if applicable.
**21. Annotation process**	Detail the annotation process, including annotator background, roles, training, as well as the number of annotators and test rounds.
**22. Annotation guidelines**	Describe the annotation guidelines used, including how they were developed, tested, and refined. Explicitly state whether the guidelines are publicly available.
**23. Annotation tools**	Mention any software used to support the guideline development and annotation process.
**24. Inter-annotator agreement**	Describe how annotation consistency was measured and report the agreement metrics used as well as how disagreements were handled.
**Outcome**	
**25. Performance**	Provide detailed performance metrics for each information type as well as aggregated over all types, such as precision, recall, and F1 score. Justify each metric used. Explicitly state the calculation formula for each metric. For continuous variables, show the risk for outliers (eg, scatterplots). Describe the aggregation method used for calculating averaged performance measures. If available, compare with human-level and baseline performance. Describe (manual) error analysis results. For classification tasks, provide confusion matrices.
**26. Statistical validation**	Describe any statistical validation procedures applied.
**27. External validation**	If applicable, report results of validating the information extraction algorithm on an independent dataset from an institution than that of training/test data and detail the dataset’s characteristics. If external validation is not possible, it should be acknowledged as limitation.
**28. Data availability**	Indicate whether and how the datasets used in the study are publicly available, accessible upon request, or not available (including the rationale).
**29. Source code availability**	Explicitly state whether the source code is made available. If yes, provide it via open repositories (eg, Zenodo). If applicable, provide links to trained models (either via upload to a platform or direct download).

Explanation, elaboration, and examples for each item are provided in the Explanation and Elaboration document, provided as supplementary material.

CINEX is provided both as a human- and machine-readable guideline and hosted on a publicly available website (https://cinex-guideline.org), featuring a fillable checklist, downloadable templates, and version history. To ensure that the guideline remains current, applicable, and manageable, we established a structured update process.

Feedback is collected continuously via the website’s public GitHub repository and triaged by the CINEX executive board. Proposed changes are screened for relevance and evaluated in periodic review cycles based on criteria such as empirical support, generalizability, and impact on usability.

Updates undergo a public commenting phase before final approval by the executive board and are released on a regular 3-year cycle or earlier if major methodological advances occur. All changes are versioned and documented to ensure transparency and reproducibility.

## Discussion

### Summary of key findings

In this study, we developed the CINEX guideline, a set of minimum reporting requirements for studies on clinical IE. With its 29 checklist items grouped into 5 categories (1) information model, (2) architecture, (3) data, (4) annotation, and (5) outcome, CINEX is designed to be applicable to all IE tasks and implementation paradigms in the clinical domain.

The heterogeneity observed in reporting practices across clinical IE studies is indicative of more than just stylistic differences. This highlights fundamental discrepancies in how key methodological elements are defined, documented, and justified, a pattern identified in radiology-focused IE studies^22^ and likely extending more broadly. This lack of transparency hinders the interpretation of results, restricts meaningful comparisons between approaches, and undermines reproducibility. In particular, Kolbinger et al^20^ emphasize the need for common standards that address task-specific methodological gaps. In clinical IE, these gaps are evident due to the pivotal role of domain-specific information models and annotation processes. Despite their critical impact on validity, these processes are often underreported. In direct response to this evidence-based need, CINEX aims to improve the transparency, comparability, and interpretability of clinical IE research, providing minimum reporting requirements tailored to the methodological realities of clinical IE.

### Relation to existing reporting guidelines

CINEX is specifically designed for clinical IE. Rather than competing with general AI reporting guidelines, it complements established standards such as SPIRIT-AI,^15^ CONSORT-AI,^16^ TRIPOD-AI,^17^ and TRIPOD-LLM^18^ by addressing methodological elements that these frameworks usually only cover indirectly. While existing guidelines primarily target prediction modeling and interventional AI evaluations, the primary ‘outcome’ of clinical IE is a structured representation derived from narrative text (eg, entities, relations, events or document-level structures), rather than a prediction or decision. Consequently, the key sources of validity in IE, eg, the definition of target information types, specification of the underlying information model/ontology, annotation design and alignment between annotation and evaluation, are not fully captured by broader AI checklists. Table 1 summarizes how existing reporting guidelines cover the IE-specific reporting elements defined by CINEX.

Existing guidelines offer little guidance on reporting what is extracted (semantic scope and granularity), how it is represented (information model, schema, normalization, temporality, and context), and how ground truth is produced (annotation guidelines, annotator training, adjudication and agreement), which are essential dimensions in IE. CINEX addresses these gaps by providing reporting requirements that are tailored to the specific semantics, data dependencies, and evaluation challenges of clinical IE (eg, label ambiguity, contextual qualifiers, cross-document dependencies and metric choice in the case of class imbalance). When used alongside broader AI reporting standards, CINEX enables more comprehensive and methodologically sound reporting of clinical NLP studies.

Recent work has proposed practical guidance for reporting NLP methods,^29^ structured around 4 domains: validity, transparency, reproducibility, and safety. This work is complementary to CINEX but differs in its primary aim. It offers flexible, practice-oriented recommendations, whereas CINEX defines a minimum reporting standard intended to harmonize reporting across studies. Safety considerations such as hallucinations, bias, and potential harm are explicitly covered by item <28>, but are not a central focus of CINEX. For clinical IE, hallucination and fabrication are not only general LLM safety concerns but can result in IE-specific failures when systems extract entities, relations, or attributes that are not supported by the source text. CINEX indirectly supports the detection and interpretation of such risks through transparent reporting of data provenance, annotation procedures, evaluation strategy, error analysis, and dataset characterization; however, future iterations should consider whether a dedicated safety dimension is necessary.

### Implications for research, review, and clinical translation

CINEX has implications throughout the research lifecycle, from study design and reporting to appraisal and eventual clinical translation. For researchers, it provides a structured reporting framework that encourages the consistent documentation of elements critical to the validity of clinical IE (eg, target information types, information model/schema, data provenance, annotation design, and evaluation). This improves reproducibility, enables meaningful comparisons across studies and supports reuse in secondary analyses, and evidence synthesis. For peer reviewers and journal editors, CINEX offers a shared point of reference that reduces variability in assessment, makes methodological omissions easier to detect and strengthens decisions about transparency and interpretability. For clinicians, implementers, and other healthcare stakeholders, more complete reporting improves the interpretability of reported performance, clarifies the clinical meaning of extracted outputs and supports a more informed evaluation of the potential benefits, limitations, and risks in specific care settings.

By promoting transparent documentation of the information model, system architecture, data sources, annotation workflow, and evaluation strategy, CINEX enables the responsible development and safer integration of clinical IE systems into healthcare environments.^30^ Hidden dependencies, such as local documentation practices,^31^ coding conventions, language, and annotation choices, can materially affect performance and failure modes in these environments.

CINEX was intentionally designed to be task- and method-agnostic to maximize its applicability across common IE paradigms (eg, named entity recognition, relation extraction, and document-level extraction or classification) and approaches (eg, rule-based, statistical or machine learning, and large language model-based systems). Accordingly, CINEX defines a minimum reporting set that should be addressed in all studies. While some items may not be applicable to every task or method, authors are expected to explicitly state and justify any such cases. “Not applicable” responses therefore require a clear rationale, ensuring that omissions are transparent and assessable rather than implicit. This requirement aims to prevent the non-reporting of relevant information while preserving flexibility across diverse settings. To further support consistent and complete reporting, future extensions of CINEX may include approach-specific reporting templates that map subsets of guideline items to particular IE paradigms.

### Limitations of the guideline and development process

The limitations of both the eDelphi process and CINEX itself should be acknowledged. Although the eDelphi rounds included experienced participants, the panel was not fully representative of the wider clinical IE community. In addition, because coauthors participated as voting panelists, the panel was not fully independent from the manuscript author group, which may have influenced item prioritization and consensus formation. Nevertheless, 20 participants contributed to rounds one and 2, which is within the range commonly considered adequate for Delphi studies.^32^ Our approach of a Delphi panel validating predefined reporting items does not guarantee the completeness of the resulting item set, as it might be biased by the experience and preferences of the panel members. As the initial candidate items were informed by a scoping review focused on IE from radiology reports, the starting item set may have been skewed toward radiology-specific reporting needs and may have underrepresented methodological issues from other clinical domains. Similarly, de-identification and anonymization procedures were discussed by panelists but were not retained as a dedicated checklist item; they are addressed indirectly through items on ethical approval, pre-processing, and data availability, but their absence as a standalone item may limit the guideline’s specificity for studies using sensitive clinical text. Furthermore, the final consensus was reached with only 12 participants, which raises the possibility that some viewpoints were not retained throughout the process.

The recruitment strategy probably introduced geographical and networking biases. Although the panel members were from 7 different countries, 45% of participants were based in Switzerland. This distribution reflects the executive committee’s reliance on their professional and personal networks, which may have limited the inclusion of perspectives from outside these networks. In addition, eligibility and expertise were assessed through self-reporting. A more objective approach, such as screening candidates based on demonstrated experience (eg, a minimum number of clinical IE publications or contributions to shared resources), could have reduced the risk of misclassification and improved transparency in panel composition. Furthermore, important contextual information about panel composition, such as disciplinary background, primary role, and sector, was not systematically collected. This limits the ability to interpret how panel expertise maps to specific guideline domains.

Methodological constraints of the Delphi design should also be considered. Panel members were not able to discuss items during rounds 1 and 2, which may have limited opportunities to resolve misunderstandings or converge on shared interpretations of items. The choice of a strict, predefined 80 % consensus threshold simplified decision-making by categorizing items as retained, revised for reevaluation, or rejected. However, alternative approaches could have produced a more graded recommendation structure. For example, items could be stratified into tiers based on agreement (eg, strongly recommended/mandatory, essential, and context-dependent), as seen in related checklist traditions. Such an approach might have yielded a more nuanced guideline but at the cost of increased complexity and potentially reduced usability.

Even beyond the Delphi process, there are limitations to CINEX as a reporting guideline. First, despite being informed by a scoping review and international consensus, some clinical domains, languages, and healthcare settings are likely to be underrepresented, which could affect the guideline’s generalizability. Second, while CINEX specifies minimum reporting requirements, it does not prescribe methodological best practices, and compliance does not guarantee study quality. Third, CINEX has not yet been applied to an existing corpus of clinical IE studies to evaluate whether it reliably distinguishes well-reported from poorly reported work, nor has its impact on reporting completeness, reproducibility, and inter-rater reliability been formally assessed by independent users.

The usability and interpretability of individual checklist items also require further evaluation. Some items, particularly the Performance item and the distinction between the clinical information model, data model, and semantic mapping, were challenging to formulate concisely and may not be fully self-explanatory when read without the Explanation and Elaboration document. Users should therefore apply CINEX together with the accompanying Explanation and Elaboration document, which provides definitions, rationales, and examples intended to disambiguate these items.

## Conclusion

The recent LLM-enabled acceleration in clinical IE developments opens doors to multiple significant healthcare applications but also raises awareness of the limitations and risks associated with these approaches. As clinical IE systems become increasingly central to healthcare workflows, the need for clear, domain-specific reporting standards has never been more urgent for fair comparability, reproducibility, and efficient translation into clinical practice with minimal risks. The CINEX guideline for clinical IE minimum reporting requirements aims to address this need.

Through a structured, consensus-based process adhering to guideline development recommendations^21^ and involving up to 20 international experts from 4 continents over a period of almost 2 years with multiple iterations comprising a Delphi study, CINEX consolidates diverse perspectives into a practical, technology-agnostic guideline that might be applied across IE tasks, clinical domains, and methodological paradigms. Its emphasis on transparent documentation of semantic definitions, annotation procedures, and dataset characteristics supports reproducibility, facilitates meaningful comparison across studies, and enhances the interpretability of reported results for both technical and clinical audiences. Each of the 29 CINEX guideline items represents a minimum reporting requirement for studies on clinical IE. Some might not be relevant in every clinical IE study and shall be indicated as such if needed. CINEX is registered on EQUATOR and published through a dedicated website in human- and machine-readable formats along with the study protocol.

CINEX complements other existing guidelines and recommendations for general AI reporting, for NLP methods reporting, or for deployable AI tools. This new guideline shall impact multiple clinical IE stakeholders. Researchers will have a reference framework supporting consistent and comprehensive reporting of all elements critical to comparability, reproducibility, and reuse in evidence synthesis. The scientific publishing industry will benefit from a reference enabling reduced variability of reviews and more transparent and interpretable decisions. Implementers in clinical settings will have access to more complete reporting helping interpret performance, potential benefits, and risks in adequate settings.

As the field continues to evolve—particularly with the emergence of multimodal models, agentic systems, and new evaluation paradigms—CINEX is intended to be a living framework, adaptable to future methodological advances and informed by ongoing community feedback. Its impact on clinical IE studies reporting consistency, completeness, and reproducibility shall be assessed.

In general, by promoting consistent, transparent, and clinically meaningful reporting, CINEX is designed to contribute to building a more robust evidence base for clinical IE and support the responsible integration of these technologies into real-world healthcare settings.

## Supplementary Material

ocag136_Supplementary_Data

## Data Availability

The CINEX guideline, checklist, templates, and version history are publicly available at https://cinex-guideline.org. Supplementary files containing the eDelphi survey instruments and item-level Delphi data are available online at https://doi.org/10.17605/OSF.IO/ATSH9.
