# Supplementary material for: The consensus-based CINEX guideline for reporting clinical information extraction studies
Source: J Am Med Inform Assoc. 2026 Aug 8;33(10):2040–50. doi: 10.1093/jamia/ocag136 (PMC13630295; doi:10.1093/jamia/ocag136)
Supplement: Supplementary file 1 [file ocag136_supplementary_data.zip › CINEX_E&E_Document.pdf]

Explanation and Elaboration of the CINEX items

Supplementary material for publication "Improving the Reporting Quality of Studies on Information Extraction From Clinical Texts: The Clinical Information Extraction (CINEX) 2026 guideline".

Version 1.0 — May 2026

Dimension: Information model

Item 1: Details of extracted information types

> Clearly list and define each type of clinical information the algorithm is designed to extract, such as entities (medications, diagnoses, procedures, or laboratory values), relations or document classes. Provide examples for each type, especially when extracting complex, multi-part types and/or concepts.

Rationale Clearly listing the types of information extracted provides readers with an overview of the extraction scope and complexity. This item catalogs *what* information types are extracted (e.g., entities, relations, attributes) and their structure (e.g., simple vs. multi-part concepts), without yet specifying the clinical definitions or frameworks that guide their interpretation (covered in Item 2). Understanding the types and granularity of extracted information is essential for assessing the study's relevance, comparing approaches across studies, and evaluating the technical difficulty of the extraction task. Concrete examples help readers understand what is extracted and at what level of detail, particularly for complex or multi-part concepts that may require sophisticated extraction methods.

Summary of key elements to address

- **Information types**: List all types of information extracted (entities, relations, attributes, document-level classifications) with brief descriptions of each type.
- **Entity types**: Name each entity type extracted (e.g., medications, conditions, procedures, laboratory tests, anatomical sites) with a short description of what constitutes each type.
- **Granularity**: Specify whether entities are atomic (single concept like 'diabetes') or complex (multi-part such as 'stage IV metastatic lung cancer with pleural effusion').
- **Attributes and modifiers**: List attributes extracted for entities (e.g., negation, temporality, certainty, subject, severity, laterality) and specify which entity types they apply to.
- **Relations**: If applicable, name the types of relations extracted between entities (e.g., TREATS linking medications to conditions, CAUSED_BY linking symptoms to diseases).
- **Quantitative information**: Describe how numerical values, measurements, and units are captured (e.g., laboratory results as test name + value + unit, medication dosages).
- **Examples:** Provide concrete examples for each information type, especially for complex or ambiguous cases.

Examples

- **Example 1: Entity extraction with attributes**

  "We extracted two types of clinical conditions from discharge summaries: chronic obstructive pulmonary disease and pneumonia. Each condition was extracted with two attributes:

  - Negation (boolean): whether the condition is present (false) or absent (true)
  - Temporality: current or past

  Example from text: 'Patient with chronic obstructive pulmonary disease. Episode of pneumonia one year ago.'

  Extracted information:

  - 'chronic obstructive pulmonary disease': negation=false, temporality=current
  - 'pneumonia': negation=false, temporality=past

- **Example 2: Complex multi-part entities and relations**

  "We extracted medications, conditions, and the treatment relationships between them. Medications were extracted as multi-part entities with two attributes: drug name and dosage.

  Example from text: 'Started on metformin 1000mg twice daily for diabetes management.'

  Extracted information:

  Entity 1: Medication

  - Drug name: metformin
  - Dosage: 1000mg twice daily

  Entity 2: Condition = 'diabetes'

  Relation: (metformin, TREATS, diabetes)"

Item 2: Underlying clinical information model

Describe the underlying clinical information model or clinical framework used to guide the structuring or interpretation of extracted data, if applicable. Furthermore, justify the choice of model and its context. Describe any adaptations or assumptions made and limitations of the information model.

Rationale Clearly defining the clinical concepts being extracted is fundamental to reproducibility and comparability across studies. Without precise definitions, the same concept name may be interpreted differently by different researchers, leading to inconsistent extraction and non-comparable results. For example, "diabetes with complications" could refer to any end-organ damage, or be restricted to specific complications like diabetic retinopathy or nephropathy. Clinical frameworks address this need for precision by providing standardized definitions and structured relationships between concepts. Established frameworks such as TNM for cancer staging, BI-RADS for breast imaging findings, or GOLD classification for COPD severity come with published definitions and operational criteria that reduce ambiguity. When using such frameworks, researchers should justify their choice: why is this particular framework appropriate for the study's clinical context and purpose? When frameworks are adapted, the rationale for adaptations must be explicit, along with any assumptions made and limitations introduced. Not all extractions are guided by established clinical frameworks. When no framework exists or applies, researchers must still define what constitutes each extracted concept, including inclusion and exclusion criteria and handling of borderline cases. This item focuses on the conceptual and clinical structure guiding extraction—"what clinical concepts should be extracted, how are they defined, and how are they organized?"—which is distinct from semantic coding (Item 4). For example, Item 2 would describe that extraction follows the Charlson Comorbidity Index framework, which includes "diabetes with end organ damage" as one of its comorbidity categories. The clinical definition from Charlson specifies that this category includes diabetes with documented retinopathy, neuropathy, or nephropathy. Item 4 would then describe how extracted text mentions are mapped to standard ICD-10 codes such as E10.2 (Type 1 diabetes mellitus with renal complications), E10.3 (Type 1 diabetes mellitus with ophthalmic complications), or E10.4 (Type 1 diabetes mellitus with neurological complications) (non-exhaustive list). Every information extraction study has a clinical purpose that fundamentally shapes what should be extracted and how it should be structured. The clinical purpose should be explicitly stated, as it provides the rationale for framework choice and helps readers assess whether the extracted information is fit for its intended use.

Summary of key elements to address

- **Clinical purpose:** State the intended use of the extraction and how it shaped the framework choice
- **Framework identification:** Name the framework with version, or explicitly state if no framework is used
- **Framework justification:** Explain why the chosen approach is appropriate for the study's clinical purpose and context
- **Adaptations and extensions:** Document modifications to standard frameworks with justification

- **Clinical definitions:** Provide clear definitions for each extracted concept, with inclusion/exclusion criteria
- **Limitations:** Acknowledge known limitations of the clinical information model in the context of the current extraction task (e.g., ambiguities in category definitions)

Examples

- **Example 1: Established framework for comorbidity extraction**

  "The clinical purpose of our study is to predict 30-day hospital readmission risk following intensive care discharge. We structured comorbidity extraction according to the Charlson Comorbidity Index (CCI) as described in the original article, chosen because it is widely validated for predicting outcomes and allows comparison with published literature. CCI defines 19 weighted categories: myocardial infarct (weight=1), congestive heart failure (1), peripheral vascular disease (1), cerebrovascular disease (1), dementia (1), chronic pulmonary disease (1), connective tissue disease (1), ulcer disease (1), mild liver disease (1), diabetes (1), hemiplegia (2), moderate or severe renal disease (2), diabetes with end organ damage (2), any tumor (2), leukemia (2), lymphoma (2), moderate or severe liver disease (3), metastatic solid tumor (6), and AIDS (6). For each patient, we extract presence/absence of each condition from discharge summaries. As an example, 'diabetes with end organ damage' is defined as diabetes with documented retinopathy, neuropathy, or nephropathy. We made no modifications to the standard CCI categories or definitions to maintain comparability with other studies.

- **Example 2: Unstructured extraction without established framework**

  "The clinical purpose of our study is to characterize the cardiovascular risk profile of patients admitted to our cardiology ward to inform care pathway optimization. We extract three key clinical concepts from admission notes that are not part of an established framework: type 2 diabetes mellitus, hypertension, and active smoking. We defined each concept with explicit clinical criteria to ensure reproducibility. 'Type 2 diabetes mellitus' is defined as documented diagnosis of type 2 diabetes, or current treatment with oral antidiabetic agents or insulin for type 2 diabetes, excluding type 1 diabetes and gestational diabetes. 'Hypertension' is defined as documented diagnosis of hypertension, or current treatment with antihypertensive medications. 'Active smoking' is defined as documentation of current cigarette smoking at admission, including number of pack-years when available, excluding former smokers who quit more than 1 month prior. These definitions were chosen because they align with our clinical purpose of identifying modifiable cardiovascular risk factors. Key limitations: the distinction between active and recent former smoking (less than 1 month) may be inconsistently documented."

Item 3: Data model

> Describe the underlying data model used to guide the structuring or interpretation of extracted data, if applicable.

Rationale The data model defines the technical structure and organization of extracted information: how concepts are represented, stored, and related to each other in a computational format. While Item 4 addresses semantic coding (terminologies like SNOMED CT), Item 3 focuses on structural organization (database schemas, message formats).

A clearly specified data model is essential for interoperability, reproducibility, and downstream use of extracted data. Different models offer different capabilities: some prioritize standardized exchange formats (e.g., HL7 FHIR), others focus on research-oriented analytics (e.g., OMOP Common Data Model), while some provide flexible document structures (e.g., JSON or XML schemas). The choice affects how easily the information can be integrated with existing systems, shared across institutions, or used for secondary analyses.

Documenting the data model allows readers to understand the technical representation, assess compatibility with their own systems, and reproduce the extraction pipeline. When multiple transformations occur (e.g., LLM extraction to JSON, then conversion to OMOP CDM), the rationale and process for each step should be described.

Summary of key elements to address

- **Data model identification:** Name the data model or schema used, including version number when applicable (e.g., OMOP CDM version 5.4, FHIR v4.3.0, custom JSON schema).
- **Rationale for choice:** Explain why this particular data model was selected for the study's purpose and context (e.g., institutional standards, interoperability requirements, analytical capabilities).
- **Structural mapping and format specifications:** Describe how extracted clinical concepts map to specific elements in the data model, including relevant technical details such as table/resource names, field names, data types, and required/optional fields (e.g., "extracted conditions stored in OMOP CONDITION_OCCURRENCE table with condition_concept_id, condition_start_date, and condition_end_date").
- **Transformation steps:** If applicable, describe any conversions between formats (e.g., LLM output in JSON converted to OMOP tables via a Python script).
- **Schema availability:** For custom data models, indicate whether the schema definition is publicly available or accessible upon request.

Examples

- **Example 1: OMOP CDM**

  "Extracted clinical concepts were structured according to the OMOP CDM version 5.4, chosen for compatibility with our institutional research database and to enable standardized observational analyses. Extracted entities were stored in the NOTE_NLP table with the following key fields: note_nlp_concept_id (mapped to SNOMED CT codes as described in Item 4), nlp_system ('llama3.2:3b'), nlp_datetime (extraction timestamp), and snippet (source text span). The NOTE_NLP table was linked to the NOTE table via note_id to preserve connection to original clinical documentation."

- **Example 2: Custom JSON schema**

  "We designed a custom JSON schema optimized for LLM-based extraction with the following structure: entity_type (string: 'condition', 'medication', 'procedure'), text (extracted mention), vocabulary (string indicating the terminology system used), code (terminology-specific identifier), negated (boolean), temporality (string: 'current', 'past', 'future'), and span (start/end character positions). This lightweight format was chosen to minimize LLM output complexity and parsing errors. Example output:

```
{
  "entities": [{
    "entity_type": "condition",
    "text": "type 2 diabetes",
    "vocabulary": "SNOMED",
    "code": "44054006",
    "negated": false,
    "temporality": "current",
    "span": {"start": 45, "end": 61}
  }]
}
```

Item 4: Semantic aspects of the extraction process

Explain how extracted entities are organized by mapping to terminologies, ontologies, or thesauri.

Rationale Semantic normalization is essential for ensuring that extracted entities are interoperable, and reusable across systems and studies. Linking extracted entities to well-established terminologies, ontologies, or thesauri (such as SNOMED CT, ICD-10-CM, RxNorm, or UMLS) helps clarify meaning, resolve ambiguity, and support consistent interpretation despite the presence of synonyms, abbreviations, and variable levels of clinical detail in free text. Explicitly recording which semantic resources are used, how mappings are performed,

and how ambiguous or unmapped entities are treated enables others to evaluate the validity, scope, and generalizability of the extraction process, as well as its suitability for downstream applications like cohort discovery and cross-dataset comparison.

Summary of key elements to address

- The terminologies, ontologies, or thesauri used to normalize extracted entities (e.g., SNOMED CT, ICD-10-CM, UMLS).
- If any, the method used for linking (e.g., exact string matching, dictionary lookup, learning-based entity linking).
- If applicable, the handling of entities that cannot be mapped to existing semantic resources.

Examples "We extract medical concepts from the notes by using the Unified Medical Language System (UMLS) [...] We use the software QuickUMLS for concept extraction [...] based on algorithm SimString to evaluate the clinical concepts from a given text. Given two strings to compare, it computes a score between 1 (perfect match) and 0 (no match at all). We retrieve the concepts by defining a threshold value with respect to the similarity of the sentences in the text and the cataloged concepts. We reduced the number of candidate concepts by increasing the threshold and restricting concepts to a selected list of semantic Type Unique Identifiers (TUIs)" [1].

Item 5: Complementing aspects of the extraction process

> If applicable, describe how uncertainty, negation, temporality, ambiguity, inconsistency and coreference resolution (e.g., patient vs. family member) is handled.

Rationale Accurately extracting information from clinical text requires more than simple entity recognition. Clinical notes often contain expressions of uncertainty (e.g. "possible pneumonia"), negation (e.g. "no history of smoking"), temporal references, ambiguous or conflicting information, and references to multiple individuals (e.g., family history vs. patient history). Properly handling these aspects ensures that the extracted data correctly reflects the patient's clinical reality and reduces the risk of errors in downstream analyses. By documenting the methods used to address these challenges, researchers enhance transparency, reproducibility, and interpretability, and provide guidance for others aiming to apply similar extraction pipelines.

Summary of key elements to address

- Methods for detecting negation (e.g., "no evidence of", "denies") and uncertainty (e.g., "possible", "likely", "suspected").

- If applicable, methods for handling temporality and timing of clinical events (e.g., "last month", "future risk").
- If applicable, methods for resolving ambiguity or inconsistency in statements across notes or within the same note.
- If applicable, methods for coreference resolution, distinguishing between the patient and others (e.g., family members).

Examples "The status of an assertion explains how a named entity (e.g. clinical finding, procedure, lab result) pertains to the patient by assigning a label such as present ("patient is diabetic"), absent ("patient denies nausea"), conditional ("dyspnea while climbing stairs"), or associated with someone else ("family history of depression"). [2]"

"We fine tuned LLama3.1-8B model with the i2b2 assertion training dataset using LoRA fine-tuning approach without quantization. [...] We explicitly included a detailed description of each assertion status to evidently improve performance. Additionally, we replaced the term Present with Confirmed which yielded better results, likely due to improved clarity and alignment with the task's semantics. [...] across multiple assertion categories (12 categories, more than what is covered in this study) including Present, Past, Possible, Absent, Hypothetical, Family, Someone Else, Planned, Conditional, Confirmed, Negative, and Suspected. [2]"

Dimension: Architecture

Item 06: Task

> Describe the primary information extraction task(s) as well as additional steps performed as part of the study, such as document classification, named entity recognition (NER), relation extraction, template filling, or summarization. For complex workflows (e.g., agentic behaviour, multi-step pipelines), describe each step in detail.

Rationale Clearly defining the NLP task(s) under investigation is essential for understanding the study's objectives, methodological choices, and evaluation strategy. Ambiguity about what the model is intended to achieve can hinder reproducibility and comparability across studies. Specifying the task type and its operational definition, that is, what the system is expected to identify, extract, or predict, enables readers to situate the work within the broader NLP landscape and understand its clinical relevance. Moreover, many studies use multi-step pipelines or multi-task learning frameworks; in such cases, each subtask should be described to clarify dependencies and information flow. Transparency about the task also facilitates evaluation alignment: metrics such as F1-score, accuracy, or BLEU may have different interpretations depending on the underlying task formulation. Providing a clear description of the task ensures that performance results are meaningful and comparable.

Summary of key elements to address

- The primary NLP task(s) addressed (e.g., NER, relation extraction, text classification, summarization, etc).
- A definition of each task, what information or concept is being extracted, predicted, or classified (e.g. treatment, disease, liver size, etc).
- If applicable, clarification of the scope and purpose of each task within the study (e.g., preprocessing, final prediction, interpretability enhancement).

Examples "In this work, we explore two perspectives for NER : as information extraction and as word representation. For the first, named entities are considered as a sequence classification problem, for which we propose a baseline method using the conditional random fields (CRF) framework. For the latter, our methodology is based on different deep neural language models derived from the BERT architecture. These methods were used to extract named entities in subtask 1 - symptoms and signs, and pathology - and subtask 2 - anatomy, dose, exam, mode, moment, substance, treatment, and value of the DEFT Information extraction task" [3].

Item 7: Implementation

> Detail the implementation strategy used to develop the algorithm, including any specific frameworks, pipelines, or approaches. When using existing software, include version specifiers and parameters. If applicable, depict the complete machine learning system architecture including its components. For LLMs, provide the model card.

Rationale The reproducibility of computational research relies heavily on the transparency of the software environment and architectural design. Even minor differences in software packages or versions can lead to different results despite using identical datasets and methods. Explicitly naming frameworks (e.g., PyTorch, TensorFlow) and their specific versions allows independent verification and reduces technical barriers for future researchers.

Beyond the software stack, visualizing the system architecture is needed for complex, multi-stage pipelines common in clinical NLP, where data may flow through distinct modules for pre-processing, entity recognition, and relation linking. For LLMs, referring to a model card is now a standard; it provides context regarding the training data of the base model, intended use cases, and inherent limitations that significantly impact clinical safety and bias.

Summary of key elements to address

- Software frameworks and libraries: List all major libraries used (e.g., scikit-learn, spaCy, PyTorch) with exact version numbers.
- Base models and checkpoints: For transfer learning or LLMs, specify the exact pre-trained model checkpoint used (e.g., `bert-base-uncased`, `Llama-2-70b-chat-hf`).
- Pipeline architecture: Describe the flow of data through the system components.
- Architecture diagram: Include a visual representation (e.g., flow diagram) of the model architecture or processing pipeline if the workflow involves multiple distinct stages.
- Model card reference: For LLMs, cite the original model card or provide an equivalent summary of the model's lineage and characteristics.

Examples

- *Traditional deep learning*: The system was implemented using PyTorch v1.13.1 and the Hugging Face Transformers library v4.26.0. We utilized the `BioClinicalBERT` checkpoint as the encoder backbone. The architecture consists of a shared encoder followed by two task-specific heads: a conditional random field (CRF) layer for NER and a biaffine classifier for relation extraction, as depicted in Figure X.

- *LLM-based extraction*: We employed `GPT-4-turbo` (version `gpt-4-0125-preview`) accessed via the OpenAI API. The extraction pipeline was orchestrated using LangChain v0.1.0 to manage prompt templates and context retrieval. To ensure reproducibility of the prompt engineering process, all prompt templates are version-controlled. Characteristics of the base model, including training cut-offs and safety alignments, are detailed in the referenced model card.

Item 8: Pre-processing

> Describe text normalization or cleaning steps performed prior to model input, including the rationale and details of the approach taken. When using existing software, include version specifiers and parameters.

Rationale Pre-processing is a critical step in transforming raw clinical text into a format suitable for computational models. Clinical narratives often contain inconsistencies such as spelling variations, abbreviations, ungrammatical sentences, punctuation artifacts, and formatting irregularities. These can negatively impact downstream tasks like tokenization, feature extraction, and model performance. Normalization and cleaning procedures help ensure that models learn from meaningful linguistic patterns rather than noise; however, these transformations can also introduce bias or remove clinically relevant information if applied inappropriately. Therefore, pre-processing decisions must be meticulously documented, including software versions, parameter settings, and the rationale behind chosen operations. Transparent reporting enables reproducibility, facilitates comparison across studies, and prevents hidden sources of methodological variability.

Summary of key elements to address

- Specify all normalization steps (e.g., lowercasing, removal of special characters, handling of punctuation).
- Describe how domain-specific elements (e.g., abbreviations, clinical jargon) were treated.
- Report any tokenization or sentence segmentation methods applied.
- Include details of software tools used (name, version, parameters).
- Explain the rationale for each step (e.g., improving consistency, reducing noise).

Examples

- The pre-processing module normalizes the text by switching case to lowercase (Diabète et 'Behçet' → diabète et 'behçet'), removing diacritics (diabète et 'behçet' → diabete et 'behcet'), uniforming quotes and apostrophes (diabete et 'behcet' → diabete et "behcet")

- We tokenized words and corrected the spelling using the Peter Norvig algorithm (Norvig, 2016) and the CyHunSpell Python package [4].

- Preprocessing steps included extracting text from PDF files and segmenting the content into manageable chunks using a semantic chunker. The semantic chunker was specifically used to ensure that the chunks maintained semantic coherence, a critical requirement to minimize hallucinations during information extraction [5].

- URLs embedded in the text were preserved, and HTML tags were intentionally retained to maintain the structural integrity of web-based content [6].

Item 9: Hyperparameters

> List all hyperparameters used during model training and provide their values and selection process (manual, grid search,etc.). For in-context learning, provide the exact prompts used and describe the prompt development process.

Rationale Hyperparameters, i.e., configurations set before learning begins, directly govern the complexity, convergence, and final performance of a model. Reporting their exact values (e.g., learning rate, batch size, dropout rate) is a cornerstone of reproducibility, as even minor variations can lead to significantly different outcomes. However, reporting the values alone is insufficient. The validity of these parameters depends entirely on the method and, but most importantly, on the data used to select them.

A common methodological failure in machine learning is test set leakage, where researchers iteratively tune parameters based on test set performance, effectively overfitting the model to the evaluation data. To demonstrate the rigor of the development process, it is essential to specify the optimization strategy (e.g., grid search, Bayesian optimization) and explicitly state which data split (e.g., validation set, development set) was used for tuning.

In the context of LLMs, these principles extend to prompt engineering. For models using in-context learning, the prompt itself, including its instructions and few-shot examples, acts as a complex hyperparameter that conditions the output of the model. Because prompts are highly sensitive to wording, the exact templates must be reported. Furthermore, to prove that the prompts generalize well, authors must clarify that prompt iteration and refinement were performed on a development set, rather than the final test set.

Summary of key elements to address

- Training hyperparameters: List values for learning rate, batch size, epochs, weight decay, dropout, and optimizer-specific settings.
- Inference hyperparameters: For generative tasks, specify temperature, top-p (nucleus sampling), top-k, repetition penalty, and maximum token length.
- Tuning data source: Explicitly state which data split was used for optimization (e.g., "tuned on the 10% validation split", "selected via 5-fold cross-validation") to confirm that the test set was blinded during development.
- Optimization strategy: Describe the method used to select these values (e.g., manual tuning, random search, grid search) and the search space explored.
- Prompt specifics: Provide the full text of system prompts and user prompts. If few-shot learning was used, describe the selection criteria for the examples (exemplars) and confirm they were drawn from non-test data.

Examples

- *BERT fine-tuning*: We fine-tuned the ClinicalBERT model for 4 epochs using a batch size of 32 and a learning rate of 2e-5. These values were selected via a grid search performed on the validation set (20% of the training corpus). The search space included learning rates of 1e-5, 2e-5, 5e-5 and batch sizes of 16, 32. The test set was accessed only once for the final evaluation.
- *LLM inference*: Inference was performed using Llama-3-70B with a temperature of 0.1 to minimize hallucinations and a top-p of 0.9. We applied a repetition penalty of 1.1. The maximum output token limit was set to 512.
- Prompt engineering: We utilized a 3-shot chain-of-thought (CoT) prompting strategy. The three in-context examples were randomly sampled from the training set. The prompt wording was iteratively refined based on error analysis of the development set. No test set examples were inspected during the prompt engineering phase. The exact prompt template is provided in Appendix B.

Item 10: Hardware details

Report hardware specifications (e.g., GPU device name, vRAM, cluster description, GPU hours) of training and inference environments and costs, if applicable and available.

Rationale Hardware is often treated as a sunk cost or invisible abstraction, but it fundamentally shapes research outcomes; a phenomenon described as the *hardware lottery*, where ideas succeed not because of merit but because they fit available hardware. Reporting specific accelerator types (e.g., NVIDIA A100 vs. V100) and memory limits (particularly, video RAM (vRAM)) is necessary for reproducibility. A model trained on an 80GB GPU may simply fail with out-of-memory errors on a standard 16GB card, rendering the study irreproducible for many labs.

Furthermore, distinguishing between training and inference hardware is necessary for clinical applicability. While training may require massive HPC clusters, clinical deployment often occurs on modest hospital servers or edge devices. Understanding this inference gap helps practitioners assess feasibility. Finally, reporting the computational budget (GPU hours) and carbon footprint aligns with *Green AI* principles, promoting efficiency alongside accuracy and creating accountability for the environmental cost of large-scale NLP.

Summary of key elements to address

- Compute specifications: Specify the make, model, and count of accelerators (e.g., 4x NVIDIA A100-SXM4) and CPUs used.
- Memory constraints: Explicitly report vRAM per GPU and total system RAM, as these are often the primary bottlenecks for reproduction.
- Inference environment: Detail the hardware used specifically for latency/throughput benchmarks, if different from training.
- Computational budget: Quantify the total resources consumed, typically in GPU-hours or CPU-hours.
- Environmental impact: Where feasible, estimate the carbon emissions (kgCO_2eq) using tools like CodeCarbon or CarbonTracker.

Examples

- *High-resource training*: Models were trained on a high-performance cluster node with 32x NVIDIA A100 GPUs (80GB vRAM each) interconnected via NVLink. The total system RAM was 4TB. Training the final model required approximately 450 GPU-hours. We estimated the carbon footprint to be 48 kgCO_2eq using the local grid carbon intensity.
- *Clinical inference*: While training utilized cloud-based TPU v4 pods, all inference benchmarks were conducted on a single NVIDIA T4 GPU (16GB vRAM) with an Intel Xeon CPU (2.3 GHz) to simulate a typical hospital deployment server. The average inference latency on this hardware was 120ms per report.

Dimension: Data

Item 11: Data flow

> Provide a detailed description of the data processing workflow, to report on the processing of selecting, excluding, filtering and sampling data from the originally available pool of documents and on the number of resulting documents/sentences/words/tokens for each step. The provision of a visualization is complementary.

Rationale Transparent reporting of the data flow is essential in studies involving clinical text, where datasets frequently undergo multiple processing steps (e.g., data filtering, de-duplication, sampling, or exclusion based on metadata or content) before they are used for annotation or model development. These steps can create a new source of bias as they substantially affect dataset composition, linguistic properties, and representativeness. Providing a clear description of how the initial corpus was transformed into the final analytical dataset allows readers to understand potential sources of bias, assess the reproducibility of the study, and judge whether the findings can be generalized to other settings. Since even minor preprocessing operations (e.g., sentence splitting or document merging) can influence token counts, annotation difficulty, and model performance, documenting each transformation, with associated quantitative changes, ensures scientific transparency. If possible, a flow diagram or table summarizing the number of documents or tokens at each stage further enhances clarity and facilitates comparison across studies.

Summary of key elements to address

- The initial size and composition of the dataset (e.g., number of documents, document types, time span).
- A step-by-step description of all preprocessing operations (e.g., filtering rules, exclusion criteria, de-identification, sentence segmentation).
- Quantitative counts after each step (documents, sentences, tokens, or other relevant units).
- Description of any data loss, missing data handling, or reasons for excluded items.
- If any sampling, an indication of any strategy (e.g., random, stratified) and their justification.
- Optional but recommended: a visual representation (e.g., flowchart, Sankey diagram) of the data pipeline.

Examples ”In large medical centers like the University of California, San Francisco (UCSF), about 2 million new clinical notes are generated monthly, thus steadily building the current corpus of 130 million notes (as of January 2023).

[...] The automated pipeline has 8 main modules that use our Notes extract, transform, load process during each data refresh:

1. Extract clinical notes texts and corresponding metadata from Clarity.
2. Load metadata, date offsets (for date shifting), and ID mappings (for original to surrogate ID crosswalks) extracted from the Clinical Data Warehouse into the Meta Data collection.
3. Load texts corresponding to the metadata into the Raw Text collection.
4. Load PII data extracted from Clarity into the Probes collection.
5. Create a Batch list of notes for de-identification for the current refresh. This includes the subset of notes added to Clarity and the notes that were modified since the previous refresh.
6. Run Philter V1.0 on notes in the Batch list and save the deidentified notes in the Deid Text collection.
7. Create the Meta Data View collection with only deidentified data for users by hiding original identifiers and date offsets from the metadata.
8. Transfer the de-identified clinical note texts and userfacing metadata to Information Commons

[...] We therefore used 100000 random clinical notes to train the algorithm and tested on batches of 2 million random notes [...] a total of 10M de-identified clinical notes was provided [...] FRule requires that all data from patients older than 90 years be obfuscated. For these patients, Philter V1.0 shifts all birth dates such that they appear to be at most 90 years old

[...] Small-town names (population ¡20000) in the clinical notes poses a risk for reidentification of patients from those towns. To address this issue, we created an exclude list of town names with population ¡30000 from the US census registry.

[...] Batches of 2 million notes were randomly drawn for iterative testing by ArcherHall, and 100,000 notes were randomly selected for algorithm training to capture rare edge cases of PHI.

[...] Figure 1. Schematic of Philter V1.0 pipeline. (A) Complete pipeline, (B) MongoDB automation, and (C) Philter V1.0 algorithm features” [7].

Item 12: Splitting strategy

> Show and explicitly describe the applied data splitting strategy (train, test, evaluation and/or validation set) including amount of documents/sentences/words/tokens, splitting modality (timeline, groups, etc.), and method (random, stratified).

Rationale The data splitting strategy is an important determinant of model validity, and generalizability. Clearly describing how data are divided into training, validation, and test sets allows readers to understand the experimental design and to assess the robustness of the reported results. Improper or poorly documented splits can lead to data leakage, influencing evaluation results, and thus inflating performance outputs.

This risk is especially pronounced in clinical NLP, where multiple notes may belong to the same patient. Reporting whether splits are made by patient, encounter, document, or time period is therefore essential for transparency and reproducibility. Furthermore, the splitting modality (e.g., temporal, stratified, k-fold cross-validation) often reflects the intended real-world deployment of the model. For example, a temporal split may simulate future data prediction, while a stratified split ensures balanced representation of clinical outcomes or entity types. Without explicit detail, it becomes challenging to replicate findings or fairly compare systems across studies or datasets.

Summary of key elements to address

- A clear description of the splitting scheme (e.g., training/validation/test proportions, cross-validation if any).
- The unit of splitting (e.g., document, patient, admission, sentence) and rationale for the chosen level.
- The size of each subset reported in either percentages, absolute counts (documents, sentences, tokens), or both.
- Any constraints (e.g. domain-specific or cohort-specific), such as ensuring that notes from the same patient do not appear in both training and test sets.
- If any cross-validation procedure, the number of folds.

Examples "The cohort was split into a training and testing dataset by treatment start dates, using data up to 2020 for training and from 2021 onwards for testing. The treatment start date was used to split the data to simulate data cut-offs that would be expected in a real-world deployment of CLEAR. To evaluate the similarity between the training and testing data after the date split, we analyzed the proportion of key concept mentions (unemployment, homelessness, food insecurity, substance dependence, suicidal ideation, depression, overdose) in both sets. Using a regular expression search with synonyms for each variable (Supplementary Table 14), we found minor differences in concept mentions across the datasets (Supplementary Table 15). There were 767 patients in the training dataset with 16031 unique notes and 505 patients in the testing dataset with 12319 unique notes. Combined, the testing and training datasets had a min, median, and max token lengths of 218, 1778, and 10,981, respectively" [8].

Item 13: Origin

> Identify the department(s), institution(s) and country(ies) from which the clinical texts originated. If applicable, describe which (clinical) information system data was extracted from.

Rationale Clearly documenting the department, institution, and country of origin enhances understanding and comparability of studies. Reporting guidelines for observational studies (e.g., STROBE) already emphasize describing the setting and location of data collection [9]. Clinical documentation practices vary widely between departments, hospitals, and regions; as a result, NLP systems developed on notes from one setting often do not generalize well to others. Stating the data origin allows readers to assess the context and limits of a dataset's representativeness, and to assess potential biases introduced by local documentation styles or patient populations. By explicitly detailing where and how the texts were obtained (including the clinical information system used), authors enable a more rigorous evaluation of the work's validity and applicability to other settings.

Summary of key elements to address

- The clinical setting or department from which the texts originated (e.g., emergency department, radiology unit, primary care clinic).
- The name or type of institution(s) (hospital, health system, etc.) and the country (or region) of origin. Clearly state if data stem from a single center or multiple institutions (multisite dataset).
- The clinical information system or database used to source the text, if applicable (e.g., specific EHR platform, data warehouse, or open clinical text repository).

Examples "MIMIC-III integrates deidentified, comprehensive clinical data of patients admitted to the Beth Israel Deaconess Medical Center in Boston, Massachusetts, and makes it widely accessible to researchers internationally under a data use agreement" [10].

"RadCore is a multi-institutional database of radiology reports aggregated in 2007 from three major healthcare organizations: Mayo Clinic, MD Anderson Cancer Center, and Medical College of Wisconsin. RadCore radiology reports were collected under institutional review board approval from those three organizations. There were no major differences in the formatting of chest CT radiology reports in these different organizations" [11].

Item 14: Type

> Describe the clinical text source (author type, stage in patient treatment where it is created (e.g., at discharge, during stay) and their

purpose (e.g., progress monitoring) and content (e.g., daily insights from nurses).

Rationale Different document types have distinct styles and information focuses. For example, hospital discharge summaries (often dictated by physicians) provide comprehensive overviews of a hospitalization, but their content and structure can vary greatly between institutions or clinicians [12]. In contrast, daily progress notes or nursing notes are typically shorter, focused on ongoing patient status, and may emphasize care coordination. Domain-specific reports likewise differ: a radiology report from an MRI exam will emphasize different anatomical structures and findings than an X-ray report, meaning an IE model trained on one modality may not generalize to another. Clearly documenting the text source type enables readers to understand such context and judge the applicability of the results. Failing to specify the note type (e.g., simply calling them "clinical notes") can obscure critical details.

Summary of key elements to address

- The document type(s) used (e.g., discharge summaries, progress notes, nursing notes, radiology reports, operative notes).
- The author role for each document type (e.g., attending physician, resident, nurse, radiologist, multidisciplinary team).
- The stage of the patient care pathway at which the text is created (e.g., admission, during inpatient stay, at discharge, outpatient follow-up).
- The primary purpose and target group of the text (e.g., longitudinal progress monitoring, care coordination, diagnostic interpretation, episode-of-care summarization).
- The typical content and structure of the text (e.g., narrative free text, semi-structured templates, section headers, level of detail).
- Any anatomical focus of the text (e.g., chest imaging reports, abdominal operative notes).
- If multiple text types are used, a clear description of each type and how they differ in authorship, timing, purpose, or content.

Examples "The information extraction task targeted diagnosis and procedure mentions in physician-authored discharge summaries. The discharge summaries were created at the end of an inpatient hospital stay by attending physicians and residents to summarize the full episode of care. The documents consisted of narrative free text organized with semi-structured section headers (e.g., Hospital Course, Discharge Diagnoses)."

"The information extraction task focused on identifying symptom progression and intervention mentions in nurse-authored ICU progress notes. These

notes were documented once per shift during the inpatient stay by bedside nurses to support ongoing progress monitoring and multidisciplinary care coordination. The content was predominantly unstructured narrative text describing current patient status, administered interventions, and observed clinical changes."

Item 15: Timeframe

> Describe the timeframe and (binned) time-related distribution of the dataset.

Rationale Describing the timeframe and time-related distribution of the dataset is important because they define the observational window of the dataset and determine the extent to which the data are appropriate for the analytical objectives. They facilitate the identification of temporal patterns, including long-term trends, cyclical behaviors, and anomalous events. They also enable the detection of temporal imbalances that may introduce bias into statistical or predictive models. Moreover, understanding the temporal structure informs the selection of suitable analytical techniques, particularly those designed for time-dependent data. Finally, this information supports the design of appropriate preprocessing procedures, such as addressing missing intervals or performing temporal aggregation.

Summary of key elements to address

- **Study period**: The lower and upper bounds of the study period need to be specified.
- **Periodicity**: The periodicity selected for the data collection should be clarifies
- **Practice changes**: Any changes in practice that occurred during the study period

Examples The dataset covers the period from January 2010 to December 2022, with monthly data collection. A modification in the calculation of the indicator occurred in January 2019, introducing additional variables.

Item 16: Representativeness and imbalance measures

> Describe the population the data used in the study might be representative for, including any limitations. If applicable, report the class distribution on all levels (source, sample, split) and any methods used to address class imbalance.

Rationale This item emphasizes describing how well the study's dataset represents a broader target population and noting any generalizability limits. For example, data drawn from a single hospital or demographic group may not generalize widely [13]. A representative sample means that findings could reasonably apply to the intended population, so acknowledging any narrow sampling (e.g., single-center data or specific patient subgroups) is crucial [14]. Additionally, authors should report the class distribution (e.g., outcomes or label frequencies) at every level (e.g., in the original source data and in the study's analyzed sample), and separately for every data subset/split. Imbalanced class distributions can bias model training and evaluation, as models may over-focus on majority classes and under-detect minority cases [15]. Stating the proportion of each class and any techniques used to address imbalance (such as stratified sampling, oversampling minorities, undersampling majorities, or class-weighted loss functions) is therefore important.

Item 17: Examination and medical specialty

> If applicable, describe the examination and medical specialty the document corresponds to.

Rationale Clinical documents can vary in structure, terminology, or informational content across medical specialties. Each specialty follows specific documentation practices, and the level of clinical detail can differ markedly from one type of report to another. For example, certain surgical notes can focus primarily on the procedure itself rather than on postoperative complications, while radiology reports often follow highly standardized formats. Similarly, the length and granularity of the documentation differ between brief follow-up consultations and intensive care hospitalization notes. Medical terminology further introduces complexity through properties such as synonymy (multiple terms referring to the same concept), polysemy (a single term with several meanings) and context-dependent imprecision. These linguistic characteristics contribute to the ambiguity and implicitness of clinical language. Clearly identifying the examination type and medical specialty is therefore essential to enable accurate interpretation of datasets and to ensure transparent reporting in clinical information extraction studies.

Summary of key elements to address

- **Type of examination:** Identify the nature of the clinical examination or documentation (e.g., imaging, laboratory tests, physical exam, procedure note, specialist consultation).
- **Medical specialty:** Name the specialty or clinical service associated with each type of document (e.g., cardiology, general surgery, neurology).
- **Multiplicity:** If multiple specialties or examination types are included, list all and describe their distribution (counts or percentages).

- **Terminology:** Indicate whether a specific terminology (or several) is used in the documents.

- **Implications for information extraction:** Discuss how specialty or examination-specific characteristics may influence extraction complexity, terminology, or generalizability.

Examples *First example* The data set included cardiology consultation notes (42%), gastrointestinal surgery operative reports (48%), and radiology imaging reports (10%). Each document was labeled according to its corresponding examination type and medical specialty. Surgical reports also contained procedure codes formatted according to the *ICD-10-PCS* classification. Differences in narrative density, structure, and terminology across specialties were taken into account when designing the extraction schema and assessing system performance, ensuring that the results reflect the domain-specific variability of clinical documentation.

Second example Clinical information such as ECG findings or physical examination details were extracted from two sources: progress notes and consultation reports.

Item 18: Language

> Specify the language(s) of the clinical texts used for algorithm development and evaluation. Describe any manipulation of the original data (e.g. translation).

Rationale The performance and generalizability of algorithms, particularly those based on NLP, are highly dependent on the language of the source text. An algorithm trained on clinical notes in one language may perform poorly or fail entirely on text in another.

Known as the Bender rule: "Do state the name of the language that is being studied, even if it is English. Acknowledging that we are working on a particular language foregrounds the possibility that the techniques may, in fact, be language specific. Conversely, neglecting to state that the particular data used were in, say, English, gives a false veneer of language-independence to the work" [16].

Reporting the specific language (e.g., Spanish, English, Chinese) and any relevant variant (e.g., German vs Swiss German) is essential for readers to understand the applicability of the methods and results to their own data. Furthermore, any manipulation of the text, such as machine translation, is a critical preprocessing step that can introduce errors or alter clinical nuance. Transparency about these steps is fundamental for study reproducibility and for correctly interpreting the algorithm's performance.

Summary of key elements to address

- The specific language(s) of the clinical texts used.
- The country or region of origin, if relevant for dialect or terminology.
- A clear statement on whether any language manipulation (e.g., translation) was performed.
- If manipulation occurred, a description of the methods or tools used is required.

Examples "In our study, we use DeepL Pro to translate Spanish documents into French. This step has been applied to CANTEMIST. However, for DISTEMIST, we opt to directly use the semi-automatically translated French texts provided by the original authors" [17].

Item 19: Ethical Approval

> State whether the study received ethics committee approval and provide the approval reference number if applicable.

Rationale Research involving clinical texts uses sensitive patient health information. Ethical oversight is paramount to ensure that patient privacy is protected, data is handled securely, and the research is conducted responsibly. Stating whether the study was approved by an ethics committee, such as an Institutional Review Board (IRB), a Research Ethics Board (REB), or a Regional Ethics Committee, provides assurance to readers, reviewers, and journal editors that these principles have been upheld. Including the approval reference number adds a layer of accountability and transparency, allowing the approval to be verified if necessary. This declaration is a critical component for establishing the trustworthiness and ethical integrity of the research.

Summary of key elements to address

- A clear statement on whether ethical approval was obtained.
- The full name of the approving ethics committee or board.
- The protocol or reference number assigned by the committee, if applicable.
- Details of any specific provisions, such as a waiver of informed consent.

Examples "This study is part of the Smart and Ultrafast Breast MRI (SUBREAM) project funded by the Swiss Cancer Research (KFS-5460–08-2021-R) and approved by the Geneva Cantonal Ethics Committee (CCER) (Project-ID: 2019-00716). Informed consent was obtained from each patient for the re-use of anonymized breast imaging reports and MRI examinations" [18].

Dimension: Annotation

Item 20: Annotation Approach

> Describe whether the textual annotation method is manual, rule-based, machine learning-based, or hybrid (i.e., if automated annotation was combined with manual annotation), including its sequence. Describe the applied tagging format, if applicable.

Rationale The textual annotation approach should specify whether the method used is manual, rule-based, machine learning–based, or hybrid, as well as the sequence in which these techniques are applied. This information is essential, as medical reports often contain considerable imprecision, and the performance of annotation methods varies substantially depending on their nature. Fully manual annotation, when performed by domain experts, typically provides high precision but is time-consuming and may exhibit variability depending on the annotators' profiles (see Item 21). Rule-based or machine learning approaches are less time-intensive but raise questions about reproducibility, particularly in relation to the characteristics of the training set. Hybrid approaches offer an interesting compromise, combining advantages while mitigating some limitations of the individual methods. Additionally, the tagging format used (e.g., BIO, BILOU, or marker-based encodings such as #...#) directly influences model interoperability and reproducibility. Clearly specifying this format is therefore essential to enable faithful replication of the study and of the textual extraction methodology.

Summary of key elements to address

- Annotation method : specify whether the approach is manual, rule-based, machine-learning–based, or hybrid. Indicate the level of expertise of human annotators, the source of rules, or the type of models used.
- Workflow : Outline the full processing pipeline, including pre-processing. (normalization, tokenization, anonymization), application of rules (order, priority rules), post-processing (conflict resolution, entity merging), and human validation, where applicable.
- Expert rules: summarize how rules were designed, validated, and maintained.
- Models used in the machine-learning component: describe the model architecture, the characteristics of the training data, Key hyperparameters, the validation strategy, and the performance metrics.
- Role of human annotators: specify their involvement in creation of the gold standard, review of automated annotations, resolution of disagreements, quality control.

- Tagging format: state the encoding scheme(IOB2, IOBES, or inline markers)
- Reproducibility limitations: highlight potential barriers (dependency on training data, model availability, complexity of combined systems, non-standard tagging schemes...)

Examples We used a hybrid annotation strategy combining rule-based pre-annotation with a machine-learning sequence-labeling model. Clinical notes were normalized and tokenized before applying expert-defined lexical and contextual rules to identify candidate entities. These candidates were then refined by a fine-tuned BERT-based model trained on 4,200 annotated notes using early stopping and F1-score optimization. Two clinical annotators reviewed all automated predictions, created the initial gold standard, and resolved disagreements. Rules were derived from clinical guidelines and included keyword lists, negation handling, and exclusion patterns. All entities were encoded using the IOB2 tagging scheme.

Item 21: Annotation Process

> Detail the annotation process, including annotator background, roles, training, as well as number of annotators and test rounds.

Rationale The quality and reproducibility of annotated clinical datasets depend strongly on the profile and expertise of the annotators. Clinical narratives often contain implicit information, context-dependent meanings, and ambiguous expressions, making the annotator's domain knowledge and level of training critical for producing valid labels. Differences in background, such as clinically trained experts unfamiliar with annotation practices versus non-clinical annotators trained extensively for the task, can lead to substantial variation in annotation decisions. These characteristics influence not only label quality but also the structure of the annotation workflow, including the degree of supervision required, the complexity of training, and the type of calibration or quality-control procedures needed. The number of annotators, the reported inter-annotator agreement, and the methods used to resolve disagreements are equally important indicators of dataset consistency. Clear documentation of the entire annotation process (i.e., annotator background, roles, training steps, pilot rounds, and adjudication procedures) is essential for assessing data reliability and potential biases. Such transparency is necessary to ensure that results are reproducible and that other research teams can faithfully replicate the methods used.

Summary of key elements to address

- **Annotator background:** Specify the clinical or non-clinical expertise (e.g., board-certified physicians, medical students, trained biomedical annotators, lay annotators with structured training).

- **Roles and responsibilities:** Describe the distribution of tasks (primary annotation, secondary review, adjudication, quality control) and whether annotators worked independently or collaboratively.

- **Training and calibration:** Detail training materials, task-specific guidelines, calibration exercises, and any inter-annotator agreement assessments conducted before full annotation.

- **Number of annotators:** Report how many annotators contributed, including reviewers or adjudicators, and indicate the proportion of documents annotated by multiple individuals.

- **Test or pilot rounds:** Explain the structure, purpose, and outcomes of pilot annotation rounds (e.g., schema refinement, guideline adjustments, feasibility assessment).

- **Implications for dataset reliability:** Discuss how annotator characteristics and processes influence annotation quality, consistency, and potential biases.

Examples *First example* The dataset was annotated by six clinicians with experience in internal medicine and cardiology. Each document underwent two independent rounds of annotation by two primary annotators, followed by adjudication from a senior expert in cases of disagreement. Annotators participated in a three-week training program, including study of a detailed annotation guideline, pilot annotation of 100 documents, and weekly calibration meetings. Disagreements were documented and reviewed collectively to refine the guidelines. Inter-annotator agreement was assessed using Cohen's kappa. All guidelines, annotation manuals, and exemplar cases were version-controlled and made accessible to ensure transparency and reproducibility.

Second example Annotations were performed by a mixed team of medical students and trained curators. Following an initial calibration round of 30 notes, task instructions and examples were modified to address recurrent ambiguities in medication mentions. Each document was then labeled by one annotator and reviewed by a senior annotator, with complex cases escalated to a clinician for final adjudication.

Item 22: Annotation Guidelines

> Describe the annotation guidelines used, including how they were developed, tested, and refined. Explicitly state whether the guidelines are publicly available.

Rationale Annotation guidelines play a central role for ensuring the consistency and reproducibility of clinical text annotation. They provide a shared reference for annotators, by defining concepts to be labeled, specifying inclusion

and exclusion criteria and clarifying how ambiguous or implicit clinical expressions should be handled. The development of such guidelines generally requires iterative exchanges between clinical experts and annotators, with modifications introduced as uncertainties or recurrent disagreements emerge during pilot annotations. Documenting this development process is essential to allow readers to assess the quality, clarity, and methodological rigor of the guidelines. Well-constructed and thoroughly tested guidelines contribute directly to the creation of reliable and reproducible annotated datasets, and they support a consistent interpretation of the annotation task across individuals. Transparency is equally important. Clear reporting on how guidelines were created, refined, and validated allows other researchers to understand their quality, applicability, and limitations. Making guidelines publicly available enables independent assessment of the methodological choices made, facilitates replication by other teams, and supports comparability across studies in clinical natural language processing.

Summary of key elements to address

- **Content and purpose:** Briefly describe what the guidelines cover (e.g., definitions, entity boundaries, ambiguous cases).
- **Development process:** Explain how initial guidelines were created (e.g., based on literature, clinical standards, expert consensus).
- **Testing and refinement :** Indicate how guidelines were tested (pilot rounds, calibration exercises). Describe how feedback, disagreements, or annotation errors led to revisions.
- **Availability:** Clearly state whether the guidelines are publicly available and, if so, where they can be accessed.

Examples *First example* Annotation guidelines were initially drafted by two clinicians and a domain expert based on clinical NLP literature. A pilot round of 40 documents was annotated independently by three annotators to test clarity and identify ambiguous cases. Observed disagreements led to revisions in boundary rules and more explicit definitions for medication-related entities. The final version of the guidelines was used for all subsequent annotations and is publicly available.

Second example Guidelines were developed iteratively through discussions between annotators and the clinical lead. After a first calibration round, additional examples were added to clarify the treatment of negated events and temporal expressions. The guidelines are publicly available on the University website.

Item 23: Annotation Tools

Mention any software used to support the guideline development and annotation process.

Rationale Annotation tools play a central role in the creation and curation of datasets for NLP research, particularly in clinical and biomedical domains. The choice of tool can affect annotation efficiency, consistency, and data format compatibility with downstream models.

Different tools (e.g., INCEpTION, Brat, WebAnno, etc) offer distinct advantages: some support complex relations or nested entities, while others are optimized for collaborative workflows. Reporting which tools were used and how they were configured ensures transparency and reproducibility, allowing others to replicate or build upon the annotation pipeline.

Furthermore, some annotation tools may include specific features that can influence the quality or bias of annotations. Disclosing these details helps readers assess the reliability of the dataset and the validity of subsequent analyzes or model training.

Summary of key elements to address

- The name of the annotation software used.
- If relevant, specify the factors influencing the choice of tool (e.g., suitability for sensitive data management, ease of use, or compatibility with existing workflows).

Examples "We used the BRAT Rapid Annotation Tool (BRAT) [30]. BRAT is designed in particular for structured annotation (including entities, relations and attributes), and provides a native support for Chinese text. It allows flexible customization of visualizations via the configuration files. In particular, we carefully set up the color scheme for entities, so that different entity types were visually distinctive, and the corresponding semantics could be, to some extent, reflected by the entity colors. For example, the Disease entities were colored in red, whereas the Treatment entities were in green. As reported by the annotators, these configurations provided intuitive feelings of the annotation results, which helped improve the annotation efficiency. Fig. 4 shows an example document along with its medical information annotations on the BRAT platform. The extracted medical entities, relations and attributes are also listed below. This figure demonstrates the input and output formats of our medical information extraction tasks in a fairly straightforward way" [19].

Item 24: Inter-Annotator Agreement

> Describe how annotation consistency was measured and report the agreement metrics used, as well as how disagreements were handled.

Rationale Inter-Annotator Agreement (IAA) is essential for assessing the reliability and consistency of manual annotations in clinical NLP studies. High agreement indicates that the annotation guidelines are clear and that the task is well-defined, thereby reducing bias in both model training and evaluation

[20]. Commonly used IAA metrics include Cohen's kappa for two annotators [21], Fleiss' kappa for multiple annotators [22], Krippendorff's alpha for heterogeneous data types or missing annotations [23], and F1-score–based agreement measures often used in span-based or sequence labeling tasks. Reporting these metrics provides transparency about annotation quality. Additionally, describing how disagreements were resolved, such as through adjudication or consensus meetings, ensures reproducibility and highlights potential sources of variability.

Summary of key elements to address

- Specify the number of annotators and their expertise.
- Describe the annotation guidelines and training provided.
- Report the metric(s) used to measure agreement (e.g., Cohen's kappa, Krippendorff's alpha, percent agreement).
- State the level of agreement achieved and interpret its meaning.
- Explain how disagreements were handled (e.g., adjudication by a senior annotator, consensus discussion).

Dimension: Outcome

Item 25: Performance

> Provide detailed performance metrics for each information type as well as aggregated over all types, such as precision, recall, and F1-score. Justify each metric used. Explicitly state the calculation formula for each metric. For continuous variables, show the risk for outliers (e.g., scatterplots). Describe the aggregation method used for calculating averaged performance measures. If available, compare with human-level and baseline performance. Describe (manual) error analysis results. For classification tasks, provide confusion matrices.

Rationale Transparent and comprehensive reporting of model performance is critical for assessing the validity, safety, and clinical usefulness of information extraction (IE) systems [24, 25]. Because clinical IE encompasses diverse subtasks, including but not limited to named entity recognition, relation extraction, template filling, and multi-label classification, performance can vary substantially across information types, such as entity categories (e.g., diagnoses, medications), relation types (e.g., treats, causes), template slots (e.g., dosage, duration), and document-level labels. Reporting only aggregated results can obscure clinically important disparities, such as strong performance on frequent categories but poor performance on rare or safety-critical types. Per-information-type reporting should therefore be complemented by aggregated metrics, with explicit definition of the aggregation method (e.g., micro, macro, or weighted averaging), as different strategies can lead to meaningfully different conclusions [26].

To support rigorous interpretation across clinical and technical audiences, authors should justify the selection of each evaluation metric and explicitly state the calculation formula. While precision, recall, and F1-score remain central to many IE tasks, additional metrics may be more informative depending on task and data characteristics, for example *area under the precision–recall curve (PR-AUC)* for imbalanced datasets [27], Matthews correlation coefficient (MCC) for binary classification under class imbalance [28], or exact-match accuracy and span-level scores for named entity recognition and extractive question answering [29]. For continuous outputs, reporting measures such as mean absolute error, correlation coefficients, and graphical summaries (e.g., scatterplots) helps identify systematic errors and outliers relevant for assessing clinical risk.

Performance results should also be contextualized against baseline systems and, where available, human-level benchmarks such as inter-annotator agreement. Such comparisons clarify whether model predictions align with expert judgment and where discrepancies arise [30]. Manual error analysis remains essential: qualitative examination of common error types can reveal annotation ambiguities, dataset biases, or systematic model limitations not captured by summary metrics [31]. Finally, for classification tasks, confusion matrices provide an interpretable breakdown of model behavior across classes and help

identify clinically relevant misclassifications.

Summary of key elements to address

- Report performance metrics *separately for each information type* (e.g., entity categories, relation types, template slots, document-level labels).
- Provide *aggregated performance metrics* across information types and clearly specify the aggregation method used (e.g., micro-, macro-, weighted averaging).
- Justify the choice of each evaluation metric and *explicitly state the calculation formula.*
- For continuous outputs, report error measures (e.g., MAE, correlation coefficients) and *visualize outliers or systematic deviations* (e.g., scatterplots).
- Compare model performance to relevant *baseline systems* and, when available, *human-level benchmarks* such as expert annotation or inter-annotator agreement.
- Conduct and report *manual error analysis* to identify common error types, annotation ambiguities, and model failure modes.
- For classification tasks, include *confusion matrices* to provide an interpretable breakdown of predictions across classes.

Item 26: Statistical Validation

Describe any statistical validation procedures applied.

Rationale Providing a detailed description of the statistical validation procedures is essential to establishing the reliability, transparency, and reproducibility of the study. Clearly specifying the methods employed enables an assessment of the relevancy of the statistical approaches chosen and ensures the robustness of the findings attesting that the conditions required for their valid application are met. Moreover, documenting the validation procedures helps to define the limitations of the analysis by explicitly identifying potential sources of bias or methodological constraints. These elements are critical for the accurate interpretation of the results and facilitating their reproducibility in future works or real world applicability.

Summary of key elements to address

- Assumption checks: Verify and report whether the assumptions required for the chosen statistical methods were met
- Model diagnostics : evaluate how well the model fits the data

- Validation of predictive models : report procedures used to assess predictive performance
- Statistical significance : provide test statistics, p-values, and confidence intervals
- Limitations: specify any violations of assumptions or methodological constraints

Examples The linear regression model demonstrated satisfactory predictive performance. Assumptions of linearity, independence, and homoscedasticity were met, as confirmed by residual plots and the Durbin-Watson test. Model validation using 10-fold cross-validation yielded a mean squared error of 3.9 and a mean absolute error of 1.6, indicating stable predictions across folds. The model explained 71 percent of the variance (adjusted $R^2 = 0.71$), and diagnostic checks revealed no influential observations (Cook's distance ¡ 1). All predictors were statistically significant at $\alpha = 0.05$, with 95 percent confidence intervals reported.

Item 27: External validation

> If applicable, report results of validating the information extraction algorithm on an independent dataset from another institution than the training / test data and detail the dataset's characteristics. If external validation is not possible, it should be acknowledged as limitation.

Rationale External validation is critical for evaluating the robustness, reproducibility, and generalizability of models in clinical NLP. Models that perform well on internal test sets may still fail when applied to data from different institutions, regions, languages, or clinical workflows. Differences in documentation practices, terminology, clinical populations, or EHR systems can substantially affect model performance. By testing the algorithm on an independent dataset, readers can assess whether the model's performance reflects true generalizable capability rather than overfitting to local patterns. When external validation is not feasible, clearly stating this limitation helps readers understand the contextual boundaries of the model's applicability and avoid overinterpretation.

Summary of key elements to address

- A clear statement on whether external validation was performed.
- Description of the external dataset (e.g., source institution, patient population, time period, size and, if relevant, language) and its differences with the train / dev / test data.
- Performance metrics obtained on the external dataset, reported using the same measures as internal validation for comparability.

- If any, adaptations made prior to applying the model on the external dataset (e.g., label mapping, preprocessing).
- If external validation was impossible, include a statement in the limitations including reasons (e.g., no access to external data, regulatory restrictions).

Examples "4064 cleaned and anonymised radiology reports from 2014 to 2016, including CT and PET/CT reports from a tertiary care cancer hospital in India, were used to train the models in this study.

[...] We used radiology reports from the MIMIC-III Clinical Database version 1.4 as the external validation set [70,71,72]. Of the 2083180 notes, there were 522279 radiology reports. Out of these, we extracted 501 radiology reports with unique ID a ICD-9 codes corresponding to lung carcinoma, and other cancers in the chest region (ICD9 code 162 for lung carcinoma, ICD9 codes 160, 161, 163, 164, 165 for cancers of respiratory and intrathoracic organs, ICD9 codes 150 and 151 for esophageal and stomach cancers) to have disease groups similar to the internal data. 20 reports containing blank rows were discarded (Fig. 2). This external validation set had 70.86% of reports with lung carcinoma diagnosis and 29.14% of reports with other diagnoses. The data selection criteria for the MIMIC data set are described in Fig. 2. The preprocessing of this external validation set was performed using a different rule-based Python script which included the selection of relevant sections of the report, followed by lower casing and text normalization (Supplementary material, Appendix 1).

[...] Without oversampling, the F1 scores at 95% CI for Bi-LSTM_simple, Bi-LSTM_dropout and BERT were 0.89, 0.90, and 0.86; with oversampling, the F1 scores were 0.94, 0.94, and 0.9, on internal validation. On external validation the F1-scores of Bi-LSTM_simple, Bi-LSTM_dropout and BERT models were 0.63, 0.77 and 0.80 without oversampling and 0.72, 0.78 and 0.77 with oversampling" [32].

Item 28: Data availability

> Indicate whether and how the datasets used in the study are publicly available, accessible upon request, or not available (including a rationale).

Rationale Data availability is a cornerstone of scientific transparency, reproducibility, and collaboration. A clear data availability statement allows other researchers to understand whether they can validate the study's findings, build upon the work, or apply the methods to the same dataset. While the sharing of raw clinical text is often restricted due to patient privacy regulations and institutional policies, it is crucial to address this explicitly. Providing a clear rationale for why data is not available manages expectations and demonstrates that the authors have considered their responsibilities regarding data sharing. When possible, sharing derivative, non-identifiable data, synthetic data, or the analysis code can serve as a clear alternative to enhance reproducibility.

Summary of key elements to address

- A direct statement on whether the dataset is publicly available, available upon request, or not available.
- If publicly available, the name of the repository and a persistent identifier or direct link (a DOI linking to the data repository or URL).
- If available upon request, the conditions for access and a point of contact (e.g., an online form or an email address).
- If not available, a specific and clear rationale (e.g., patient privacy concerns or institutional policy).
- Information on the availability of related materials, such as data preprocessing code.

Examples "The Stanford MOUD Cohort Dataset used in this study contains identifiable protected health information and, therefore, cannot be shared publicly. Stanford University investigators with appropriate IRB approval can contact the authors directly regarding data access. [...] The code used to run CLEAR and reproduce results can be found at https://github.com/iv-lop/clear" [8].

Item 29: Source Code Availability

> Explicitly state whether the source code is made available. If yes, provide it via open repositories (e.g., Zenodo). If applicable, provide links to trained models (either via upload to a platform or direct download).

Rationale Transparent reporting of source code availability is essential for ensuring the reproducibility, verifiability, and reusability of clinical IE studies. Making code publicly accessible allows other researchers to inspect implementation details, replicate results, identify potential sources of error, and adapt methods to new clinical contexts. Open code also facilitates peer review and accelerates methodological progress by enabling the community to build on prior work rather than reimplementing systems from scratch. Releasing trained models, when permissible, further supports reproducibility and enables benchmarking across datasets and settings.

To promote sustainable and accessible research practices, code and models should be shared through stable, citable repositories (e.g., GitHub with Zenodo archiving) that ensure long-term availability and versioning. When code or models cannot be shared due to privacy, licensing, or institutional constraints, authors should clearly state the reasons and describe alternative means for verification (e.g., synthetic examples, pseudocode, or containerized environments). Clear documentation of code availability supports transparency, aligns

with FAIR research principles [33], and increases the clinical impact and scientific value of IE systems, particularly given evidence that code availability statements are frequently incomplete or non-compliant in practice [34].

Summary of key elements to address

- Clearly state whether the source code is available.
- Provide stable, citable links to open repositories (e.g., GitHub, Zenodo, GitLab).
- Specify the version of the code used for the experiments (e.g., commit hash, release tag).
- If applicable, share trained models through accessible platforms (e.g., Hugging Face Hub, Zenodo) or provide direct download links.
- Describe any restrictions or reasons for not sharing code or models (e.g., licensing, privacy constraints).
- Provide documentation or instructions for reproducing the experimental setup (e.g., environment files, container images).
- Ensure long-term accessibility through archiving or versioning practices.

References

[1] J. Zaghir, J. F. Rodrigues-Jr, L. Goeuriot, S. Amer-Yahia, Real-world Patient Trajectory Prediction from Clinical Notes Using Artificial Neural Networks and UMLS-Based Extraction of Concepts, Journal of Healthcare Informatics Research 5 (4) (2021) 474–496. doi:10.1007/s41666-021-00100-z.
URL https://link.springer.com/10.1007/s41666-021-00100-z

[2] V. Kocaman, Y. Gul, M. A. Kaya, H. U. Haq, M. Butgul, C. Celik, D. Talby, Beyond Negation Detection: Comprehensive Assertion Detection Models for Clinical NLP, version Number: 1 (2025). doi:10.48550/ARXIV.2503.17425.
URL https://arxiv.org/abs/2503.17425

[3] J. Copara, J. Knafou, N. Naderi, C. Moro, P. Ruch, D. Teodoro, Contextualized French Language Models for Biomedical Named Entity Recognition, in: R. Cardon, N. Grabar, C. Grouin, T. Hamon (Eds.), Actes de la 6e conférence conjointe Journées d'Études sur la Parole (JEP, 33e édition), Traitement Automatique des Langues Naturelles (TALN, 27e édition), Rencontre des Étudiants Chercheurs en Informatique pour le Traitement Automatique des Langues (RÉCITAL, 22e édition). Atelier DÉfi Fouille de Textes, ATALA et AFCP, Nancy, France, 2020, pp. 36–48.
URL https://aclanthology.org/2020.jeptalnrecital-deft.4/

[4] M. M. Van Buchem, O. M. Neve, I. M. J. Kant, E. W. Steyerberg, H. Boosman, E. F. Hensen, Analyzing patient experiences using natural language processing: development and validation of the artificial intelligence patient reported experience measure (AI-PREM), BMC Medical Informatics and Decision Making 22 (1) (2022) 183. doi:10.1186/s12911-022-01923-5.
URL https://bmcmedinformdecismak.biomedcentral.com/articles/10.1186/s12911-022-01923-5

[5] A. M. Garcia-Carmona, M.-L. Prieto, E. Puertas, J.-J. Beunza, Leveraging Large Language Models for Accurate Retrieval of Patient Information From Medical Reports: Systematic Evaluation Study, JMIR AI 4 (2025) e68776. doi:10.2196/68776.
URL https://ai.jmir.org/2025/1/e68776

[6] D. Baur, J. Ansorg, C.-E. Heyde, A. Voelker, Development and Evaluation of a Retrieval-Augmented Generation Chatbot for Orthopedic and Trauma Surgery Patient Education: Mixed-Methods Study, JMIR AI 4 (2025) e75262–e75262. doi:10.2196/75262.
URL https://ai.jmir.org/2025/1/e75262

[7] L. Radhakrishnan, G. Schenk, K. Muenzen, B. Oskotsky, H. Ashouri Choshali, T. Plunkett, S. Israni, A. J. Butte, A certified de-identification system for all clinical text documents for information extraction at scale, JAMIA Open 6 (3) (2023) ooad045.

doi:10.1093/jamiaopen/ooad045.
URL https://academic.oup.com/jamiaopen/article/doi/10.1093/jamiaopen/ooad045/7219298

[8] I. Lopez, A. Swaminathan, K. Vedula, S. Narayanan, F. Nateghi Haredasht, S. P. Ma, A. S. Liang, S. Tate, M. Maddali, R. J. Gallo, N. H. Shah, J. H. Chen, Clinical entity augmented retrieval for clinical information extraction, npj Digital Medicine 8 (1) (2025) 45. doi:10.1038/s41746-024-01377-1.
URL https://www.nature.com/articles/s41746-024-01377-1

[9] E. v. Elm, D. G. Altman, M. Egger, S. J. Pocock, P. C. Gøtzsche, J. P. Vandenbroucke, The Strengthening the Reporting of Observational Studies in Epidemiology (STROBE) statement: guidelines for reporting observational studies, The Lancet 370 (9596) (2007) 1453–1457, publisher: Elsevier. doi:10.1016/S0140-6736(07)61602-X.
URL https://www.thelancet.com/journals/lancet/article/PIIS0140-6736(07)61602-X/fulltext#article_upsell

[10] A. E. W. Johnson, T. J. Pollard, L. Shen, L.-w. H. Lehman, M. Feng, M. Ghassemi, B. Moody, P. Szolovits, L. Anthony Celi, R. G. Mark, MIMIC-III, a freely accessible critical care database, Scientific Data 3 (1) (2016) 160035, publisher: Nature Publishing Group. doi:10.1038/sdata.2016.35.
URL https://www.nature.com/articles/sdata201635

[11] S. Hassanpour, C. P. Langlotz, Information extraction from multi-institutional radiology reports, Artificial Intelligence in Medicine 66 (2016) 29–39. doi:10.1016/j.artmed.2015.09.007.
URL https://linkinghub.elsevier.com/retrieve/pii/S0933365715001244

[12] K. J. O'Leary, D. M. Liebovitz, J. Feinglass, D. T. Liss, D. B. Evans, N. Kulkarni, M. P. Landler, D. W. Baker, Creating a better discharge summary: Improvement in quality and timeliness using an electronic discharge summary, Journal of Hospital Medicine 4 (4) (2009) 219–225. doi:10.1002/jhm.425.
URL https://shmpublications.onlinelibrary.wiley.com/doi/10.1002/jhm.425

[13] E. Röösli, S. Bozkurt, T. Hernandez-Boussard, Peeking into a black box, the fairness and generalizability of a MIMIC-III benchmarking model, Scientific Data 9 (1) (2022) 24, publisher: Nature Publishing Group. doi:10.1038/s41597-021-01110-7.
URL https://www.nature.com/articles/s41597-021-01110-7

[14] J. E. Rudolph, Y. Zhong, P. Duggal, S. H. Mehta, B. Lau, Defining representativeness of study samples in medical and population health research,

BMJ Medicine 2 (1), publisher: BMJ Publishing Group Ltd (May 2023). doi:10.1136/bmjmed-2022-000399.
URL https://bmjmedicine.bmj.com/content/2/1/e000399

[15] K. Ghosh, C. Bellinger, R. Corizzo, P. Branco, B. Krawczyk, N. Japkowicz, The class imbalance problem in deep learning, Machine Learning 113 (7) (2024) 4845–4901. doi:10.1007/s10994-022-06268-8.
URL https://doi.org/10.1007/s10994-022-06268-8

[16] E. M. Bender, On Achieving and Evaluating Language-Independence in NLP, Linguistic Issues in Language Technology 6 (Oct. 2011). doi:10.33011/lilt.v6i.1239.
URL https://journals.colorado.edu/index.php/lilt/article/view/1239

[17] J. Zaghir, M. Bjelogrlic, J.-P. Goldman, S. Aananou, C. Gaudet-Blavignac, C. Lovis, FRASIMED: a Clinical French Annotated Resource Produced through Crosslingual BERT-Based Annotation Projection, version Number: 1 (2023). doi:10.48550/ARXIV.2309.10770.
URL https://arxiv.org/abs/2309.10770

[18] B. Lokaj, V. Durand De Gevigney, D.-A. Djema, J. Zaghir, J.-P. Goldman, M. Bjelogrlic, H. Turbé, K. Kinkel, C. Lovis, J. Schmid, Multimodal deep learning fusion of ultrafast-DCE MRI and clinical information for breast lesion classification, Computers in Biology and Medicine 188 (2025) 109721. doi:10.1016/j.compbiomed.2025.109721.
URL https://linkinghub.elsevier.com/retrieve/pii/S001048252500071X

[19] E. Zhu, Q. Sheng, H. Yang, Y. Liu, T. Cai, J. Li, A unified framework of medical information annotation and extraction for Chinese clinical text, Artificial Intelligence in Medicine 142 (2023) 102573. doi:10.1016/j.artmed.2023.102573.
URL https://linkinghub.elsevier.com/retrieve/pii/S0933365723000878

[20] C. Oommen, Q. Howlett-Prieto, M. D. Carrithers, D. B. Hier, Interrater agreement for the annotation of neurologic signs and symptoms in electronic health records, Frontiers in Digital Health 5 (2023) 1075771. doi:10.3389/fdgth.2023.1075771.
URL https://www.frontiersin.org/articles/10.3389/fdgth.2023.1075771/full

[21] J. Cohen, A Coefficient of Agreement for Nominal Scales, Educational and Psychological Measurement 20 (1) (1960) 37–46. doi:10.1177/001316446002000104.
URL https://journals.sagepub.com/doi/10.1177/001316446002000104

[22] J. L. Fleiss, Measuring nominal scale agreement among many raters., Psychological Bulletin 76 (5) (1971) 378–382. doi:10.1037/h0031619. URL https://doi.apa.org/doi/10.1037/h0031619

[23] K. Krippendorff, Content analysis: an introduction to its methodology, third edition Edition, Sage, Los Angeles London New Delhi Singapore, 2013.

[24] R. Shankar, J. Kannan, Y. H. Tan, Q. Xu, Natural language processing techniques to detect delirium in hospitalized patients from clinical notes: a systematic review, npj Digital Medicine 8 (1) (2025) 701, publisher: Nature Publishing Group. doi:10.1038/s41746-025-02051-w. URL https://www.nature.com/articles/s41746-025-02051-w

[25] K. Blagec, G. Dorffner, M. Moradi, S. Ott, M. Samwald, A global analysis of metrics used for measuring performance in natural language processing, in: T. Shavrina, V. Mikhailov, V. Malykh, E. Artemova, O. Serikov, V. Protasov (Eds.), Proceedings of NLP Power! The First Workshop on Efficient Benchmarking in NLP, Association for Computational Linguistics, Dublin, Ireland, 2022, pp. 52–63. doi:10.18653/v1/2022.nlppower-1.6. URL https://aclanthology.org/2022.nlppower-1.6/

[26] J. Opitz, A Closer Look at Classification Evaluation Metrics and a Critical Reflection of Common Evaluation Practice, Transactions of the Association for Computational Linguistics 12 (2024) 820–836, place: Cambridge, MA Publisher: MIT Press. doi:10.1162/tacl_a_00675. URL https://aclanthology.org/2024.tacl-1.46/

[27] T. Saito, M. Rehmsmeier, The Precision-Recall Plot Is More Informative than the ROC Plot When Evaluating Binary Classifiers on Imbalanced Datasets, PLOS ONE 10 (3) (2015) e0118432, publisher: Public Library of Science. doi:10.1371/journal.pone.0118432. URL https://journals.plos.org/plosone/article?id=10.1371/journal.pone.0118432

[28] D. Chicco, M. J. Warrens, G. Jurman, The Matthews Correlation Coefficient (MCC) is More Informative Than Cohen's Kappa and Brier Score in Binary Classification Assessment, IEEE Access 9 (2021) 78368–78381. doi:10.1109/ACCESS.2021.3084050. URL https://ieeexplore.ieee.org/document/9440903

[29] A. Wang, Y. Pruksachatkun, N. Nangia, A. Singh, J. Michael, F. Hill, O. Levy, S. R. Bowman, SuperGLUE: A Stickier Benchmark for General-Purpose Language Understanding Systems, arXiv:1905.00537 [cs] (Feb. 2020). doi:10.48550/arXiv.1905.00537. URL http://arxiv.org/abs/1905.00537

[30] S. B. Yoon, J. Lee, H.-C. Lee, C.-W. Jung, H. Lee, Comparison of NLP machine learning models with human physicians for ASA Physical Status

classification, npj Digital Medicine 7 (1) (2024) 259, publisher: Nature Publishing Group. doi:10.1038/s41746-024-01259-6.
URL https://www.nature.com/articles/s41746-024-01259-6

[31] E. v. Miltenburg, M.-A. Clinciu, O. Dušek, D. Gkatzia, S. Inglis, L. Leppänen, S. Mahamood, E. Manning, S. Schoch, C. Thomson, L. Wen, Underreporting of errors in NLG output, and what to do about it, arXiv:2108.01182 [cs] (Aug. 2021). doi:10.48550/arXiv.2108.01182.
URL http://arxiv.org/abs/2108.01182

[32] S. Mithun, A. K. Jha, U. B. Sherkhane, V. Jaiswar, N. C. Purandare, V. Rangarajan, A. Dekker, S. Puts, I. Bermejo, L. Wee, Development and validation of deep learning and BERT models for classification of lung cancer radiology reports, Informatics in Medicine Unlocked 40 (2023) 101294. doi:10.1016/j.imu.2023.101294.
URL https://linkinghub.elsevier.com/retrieve/pii/S2352914823001405

[33] M. D. Wilkinson, M. Dumontier, I. J. Aalbersberg, G. Appleton, M. Axton, A. Baak, N. Blomberg, J.-W. Boiten, L. B. Da Silva Santos, P. E. Bourne, J. Bouwman, A. J. Brookes, T. Clark, M. Crosas, I. Dillo, O. Dumon, S. Edmunds, C. T. Evelo, R. Finkers, A. Gonzalez-Beltran, A. J. Gray, P. Groth, C. Goble, J. S. Grethe, J. Heringa, P. A. 'T Hoen, R. Hooft, T. Kuhn, R. Kok, J. Kok, S. J. Lusher, M. E. Martone, A. Mons, A. L. Packer, B. Persson, P. Rocca-Serra, M. Roos, R. Van Schaik, S.-A. Sansone, E. Schultes, T. Sengstag, T. Slater, G. Strawn, M. A. Swertz, M. Thompson, J. Van Der Lei, E. Van Mulligen, J. Velterop, A. Waagmeester, P. Wittenburg, K. Wolstencroft, J. Zhao, B. Mons, The FAIR Guiding Principles for scientific data management and stewardship, Scientific Data 3 (1) (2016) 160018. doi:10.1038/sdata.2016.18.
URL https://www.nature.com/articles/sdata201618

[34] C. Jean-Quartier, F. Jeanquartier, S. Stryeck, J. Simon, B. Söser, I. Hasani-Mavriqi, Sharing practices of software artefacts and source code for reproducible research, International Journal of Data Science and Analytics 20 (3) (2025) 2539–2550. doi:10.1007/s41060-024-00617-7.
URL https://link.springer.com/10.1007/s41060-024-00617-7
